# Lowering the Cu-O bond energy in CuO nanocatalysts enhances the efficiency of NH_3_ oxidation

**DOI:** 10.1038/s41467-025-64415-w

**Published:** 2025-10-24

**Authors:** Lu Chen, Xuze Guan, Zhangyi Yao, Shusaku Hayama, Matthijs A. van Spronsen, Burcu Karagoz, Georg Held, David G. Hopkinson, Christopher S. Allen, June Callison, Paul J. Dyson, Feng Ryan Wang

**Affiliations:** 1 Low-Carbon Conversion Science and Engineering Center, Shanghai Advanced Research Institute, Chinese Academy of Sciences, Shanghai, China; 2 Department of Chemical Engineering, University College London, Roberts Building, Torrington Place, London, UK; 3 Yusuf Hamied Department of Chemistry, University of Cambridge, Cambridge, UK; 4 Diamond Light Source Ltd., Harwell Science and Innovation Campus, Chilton, Didcot, UK; 5 electron Physical Science Imaging Centre, Diamond Light Source Ltd., Didcot, UK; 6 Department of Materials, University of Oxford, Oxford, UK; 7 UK Catalysis Hub, Research Complex at Harwell (RCaH), Rutherford Appleton Laboratory, Harwell, UK; 8 Institute of Chemical Sciences and Engineering, École Polytechnique Fedérale de Lausanne (EPFL), Lausanne, Switzerland

**Keywords:** Heterogeneous catalysis, Sustainability, Nanoparticles, Catalytic mechanisms

## Abstract

Tuning the electronic properties of nanocatalysts via doping with monodispersed hetero-metal atoms is an effective method used to enhance catalytic properties. Doping CuO nanoparticles with monodispersed Co atoms using different reductants affords catalysts (Co_B_Cu/Al_2_O_3_ and Co_H_Cu/Al_2_O_3_) with strikingly different electronic structures. Compared to Co_H_Cu/Al_2_O_3_, the CuO nanoparticles in Co_B_Cu/Al_2_O_3_ have longer and weaker Cu-O bonds, with a lower 1*s* → 4*p*_z_ antibonding transition and higher 4*p* → 1*s* bonding transition (as demonstrated from HERFD-XANES and valence-to-core X-ray emission spectroscopy). The weaker Cu-O bonds in Co_B_Cu/Al_2_O_3_ lead to superior redox activity of the CuO nanoparticles, evidenced from *operando* XAFS and in-situ near ambient pressure-near edge X-ray absorption fine structures studies. Such superior redox properties of CuO in Co_B_Cu/Al_2_O_3_ result in a much reduced activation energy of Co_B_Cu/Al_2_O_3_ compared to Co_H_Cu/Al_2_O_3_ (40.0 vs. 63.5 kJ/mol), thus leading to an enhancement in catalytic performance in the selective catalytic oxidation of NH_3_ to N_2_.

## Abstract (web summary)

Tuning the electronic properties of nanocatalysts by doping them with uniformly dispersed hetero-metal atoms is an effective way to improve catalytic performance. Here, the authors show that weakening the Cu–O bond energy in CuO nanocatalysts boosts the efficiency of NH₃ oxidation.

## Introduction

Ammonia (NH_3_) emissions are projected to increase in the future, driven by its expanding use as a sustainable fuel, particularly in the shipping industry, as well as emissions from industrial processes^1,2^. Since NH_3_ emissions are far less regulated than fossil fuel-based emissions, they are an important driver for fine particulate matter (PM 2.5) pollution^3,4^. Hence, the selective catalytic oxidation (SCO) of NH_3_ to N_2_ is a critical and increasingly used technology for mitigating NH_3_ emissions that are detrimental to atmospheric quality^5^. This is because NH_3_-assisted selective catalytic reduction (SCR) is the most widely used technology to reduce nitrogen oxide (NO_x_) emissions from coal-fired power stations and diesel engines^6–8^. Achieving complete NO_x_ removal necessitates an excess of NH_3_, which results in NH_3_ emissions (also referred to as NH_3_ slip). The completion of an NH_3_-SCO cycle comprises several elementary reaction steps that depend on both the properties of the catalysts and the reaction conditions, and are crucial in controlling the selectivity to N_2_. Three established mechanisms for different catalysts in the NH_3_-SCO reaction include the internal SCR (i-SCR) mechanism, the imide mechanism, and the hydrazine mechanism^9–11^. Among these, the i-SCR mechanism is predominantly applied to elucidate the NH_3_-SCO reaction pathway of Cu-based catalysts^12–16^, known for their cost-effectiveness and high selectivity.

In the i-SCR mechanism, NH_3_ undergoes initial oxidation to form NO (Step 1), and subsequently, the formed NO reacts with NH_3_, yielding N_2_ (SCR, Step 2). Step 1 involves redox processes of the metal catalyst and comprises the rate-determining step in NH_3_ oxidation^16^. Supported CuO nanoparticles (NPs) exhibit high activity and selectivity for the subsequent SCR step^17–21^, but are less active for Step 1. In contrast, supported noble metals and Co_3_O_4_ NPs exhibit high activity in Step 1, but are less efficient in the SCR step (Step 2)^22–26^. This imbalance can be overcome by integrating two metals to form a bimetallic catalyst to coordinate the sequential reactions, resulting in enhanced activity and selectivity for the entire NH_3_-SCO reaction. Several bimetallic NP catalysts have been explored for the NH_3_-SCO reaction, including those based on PtCu^27–29^, AgCu^30,31^, AuCu^32^, and RuCu.^33^ Despite the high activity of noble metal-containing catalysts, their high cost and low selectivity limit practical applications. Earth-abundant CoO_x_ offers a cost-effective alternative, with the introduction of Cu into CoO_x_ improving the N_2_ selectivity of CoO_x_-based catalysts^13,34^.

$${{{\rm{S}}}}{{{\rm{tep}}}}1:4{{{\rm{N}}}}{{{{\rm{H}}}}}_{3}+5{{{{\rm{O}}}}}_{2}\to 4{{{\rm{NO}}}}+6{{{{\rm{H}}}}}_{2}{{{\rm{O}}}}$$

$${{{\rm{S}}}}{{{\rm{tep}}}}2:4{{{\rm{NO}}}}+4{{{\rm{N}}}}{{{{\rm{H}}}}}_{3}+{{{{\rm{O}}}}}_{2}\to 4{{{{\rm{N}}}}}_{2}+6{{{{\rm{H}}}}}_{2}{{{\rm{O}}}}$$

$$4{{{\rm{NO}}}}+4{{{\rm{N}}}}{{{{\rm{H}}}}}_{3}+3{{{{\rm{O}}}}}_{2}\to 4{{{{\rm{N}}}}}_{2}{{{\rm{O}}}}+6{{{{\rm{H}}}}}_{2}{{{\rm{O}}}}$$

The activity and the selectivity of bimetallic catalysts are significantly influenced by their geometric and electronic structures^35–37^. The different electronic structures lead to differences in the strength of the metal–oxygen bond, which determines the distribution of ammonia oxidation products^38,39^. Oxides with high metal–oxygen bond strengths exhibit lower rates of reaction and facilitate a high selectivity to N_2_. In contrast, metal oxides with weak metal–oxygen bond strengths lead to the formation of NO_x_ (NO and N_2_O). The unique structural features of single-site doped bimetallic NPs allow their electronic properties to be tuned more precisely than their monometallic counterparts, which provides a facile approach to modify the metal–oxygen bond strength in order to optimize both activity and N_2_ selectivity^40^. Note that it has previously been shown that the nature of the reducing agent impacts the  catalyst structure and activity^41,42^.

In this work, the electronic structure of two bimetallic catalysts, Co_B_Cu/Al_2_O_3_ and Co_H_Cu/Al_2_O_3_, prepared using NaBH_4_ and H_2_, respectively, is studied with a range of X-ray spectroscopic techniques. Despite with same chemical composition, Co_B_Cu/Al_2_O_3_ exhibits enhanced activity in the NH_3_-SCO reaction across all temperatures, surpassing the Co_H_Cu/Al_2_O_3_ catalyst. *Operando* X-ray absorption fine structure (XAFS) studies, combined with in-situ near ambient pressure-near edge X-ray absorption fine structure (NAP-NEXAFS) studies and *operando* diffuse reflectance infrared Fourier transform spectroscopy (DRIFTS), provide a detailed understanding of the electronic structure of the CuO NPs modulated by the single Co sites and the resulting impact on catalysis.

## Results and Discussion

### Synthesis and structural characterization of the catalysts

**Fig. 1:**
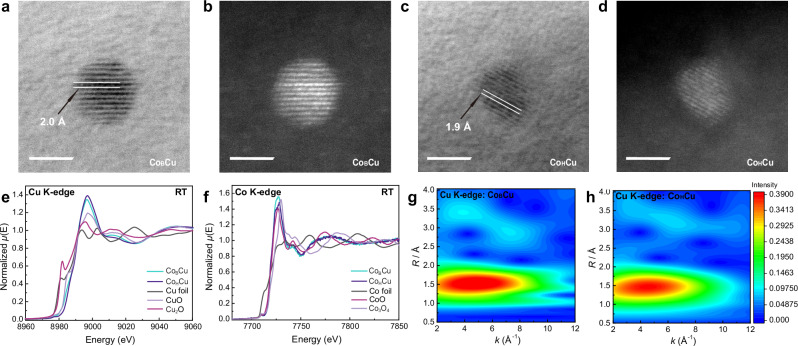
Characterization of Co_B_Cu/Al_2_O_3_ and Co_H_Cu/Al_2_O_3_. BF images (**a**) and HAADF image (**b**) of Co_B_Cu/Al_2_O_3_ (Scale bar = 2 nm); BF image (**c**) and HADDF image (**d**) of Co_B_Cu/Al_2_O_3_ (Scale bar = 2 nm); Cu K-edge HERFD-XANES (**e**) and Co K-edge XANES (**f**) of Co_B_Cu/Al_2_O_3_ and Co_H_Cu/Al_2_O_3_; 2D WT-EXAFS maps of the CuO NPs in Co_B_Cu/Al_2_O_3_ (**g**) and Co_H_Cu/Al_2_O_3_ (**h**).

The bimetallic Co_B_Cu/Al_2_O_3_ catalyst (5 wt% Cu, 0.1 wt% Co) was synthesized by reducing Cu(NO_3_)_2_^.^3H_2_O and Co(NO_3_)_2_^.^6H_2_O together with NaBH_4_. Co_H_Cu/Al_2_O_3_, with the same chemical composition, was synthesized by wet impregnation, using H_2_ as a reductant. The Cu NPs are oxidized to CuO NPs when exposed to air. As shown in bright-field (BF) images and high-angle annular dark-field (HAADF) images from scanning transmission electron microscopy (STEM), the average size of the CuO NPs in Co_B_Cu/Al_2_O_3_ is 2.6 nm, whereas in Co_H_Cu/Al_2_O_3_ the average size is 2.0 nm (Fig. 1a–d, Supplementary Figs. S1 and S2). The size of the NPs hardly changes after catalysis for both Co_B_Cu/Al_2_O_3_ (Fig. S3) and Co_H_Cu/Al_2_O_3_ (Fig. S4). The interplanar spacing of the CuO lattice in Co_B_Cu/Al_2_O_3_ (2.0 Å) is slightly longer than in Co_H_Cu/Al_2_O_3_ (1.9 Å), indicating potentially different Cu-O bond lengths in the two catalysts (Fig. 1a–d). An energy-dispersive spectrometry (EDS) map of Co_B_Cu/Al_2_O_3_ shows a uniform elemental distribution confirming the presence of Cu and Co (Fig. S5).

Electron paramagnetic resonance (EPR) spectroscopy also confirmed that the major Cu species in both catalysts comprise NPs and not monodispersed Cu sites (Fig. S6). X-ray diffraction patterns of Co_B_Cu/Al_2_O_3_ and Co_H_Cu/Al_2_O_3_ show no obvious distinctions from the Al_2_O_3_ support (PDF #10-0425) (Fig. S7a), indicative of small NPs with a uniform size distribution. H_2_-temperature programmed reduction (TPR) confirms that incorporation of Co atoms in Co_B_Cu/Al_2_O_3_ shifts the reduction temperature of CuO NPs by 34 °C (first peak) to a lower temperature (Fig. S8), indicating that Co promotes the reduction of CuO, probably via the asymmetric Co-O-Cu bond^43^. In contrast, for Co_H_Cu/Al_2_O_3_, two separate peaks indicate weaker interactions between the Cu and Co species. The broad peak in the T range of 200–300 °C of Co_B_Cu/Al_2_O_3_ in H_2_-TPR may originate from bulk CoO_x_, due to the aggregation of Co under H_2_.

The chemical environments of the Cu species in Co_B_Cu/Al_2_O_3_ and Co_H_Cu/Al_2_O_3_ are different, as determined from extended X-ray absorption fine structure (EXAFS), X-ray absorption near-edge structure (XANES), and high-energy resolution fluorescence detected - X-ray absorption near-edge structure (HERFD-XANES) measurements (Fig. 1e, f). The major Cu species in both Co_B_Cu/Al_2_O_3_ and Co_H_Cu/Al_2_O_3_ are Cu^2+^, as expected for CuO-based NPs, and the Co species in both Co_B_Cu/Al_2_O_3_ and Co_H_Cu/Al_2_O_3_ are also in the Co^2+^ oxidation state. The fitted EXAFS data reveal differences between the electronic structures of Co_B_Cu/Al_2_O_3_ and Co_H_Cu/Al_2_O_3_ (Table S1, Figs. S9–S11). Co_B_Cu/Al_2_O_3_ and Co_H_Cu/Al_2_O_3_ have a similar Cu-O coordination number (C.N.) of 3.45 ± 0.12 and 3.33 ± 0.12, respectively, and a similar Co-O C.N. of 5.74 ± 0.99 and 5.92 ± 1.09, respectively. However, Co_B_Cu/Al_2_O_3_ has a larger Cu-Cu (1) C.N. of 2.39 ± 0.48 and a Cu-Cu (2) C.N. of 1.90 ± 0.39, compared to Co_H_Cu/Al_2_O_3_ with a Cu-Cu (1) C.N. of 0.22 ± 0.21 and Cu-Cu (2) C.N. of 0. Moreover, the Cu-O bond length in Co_B_Cu/Al_2_O_3_ (1.96 Å ± 0.005) is longer than in Co_H_Cu/Al_2_O_3_ (1.93 Å ± 0.003), which is consistent with the TEM results. It is noteworthy that Co-Co bonds are not observed in either catalyst, indicative of single Co sites. Wavelet transform (WT) analysis of the EXAFS spectra leads to a 2D representation of the EXAFS (Fig. 1g, h), simultaneously revealing the signal features in both R- and k-space. The first shell peak with the most intense signal in Co_B_Cu/Al_2_O_3_ (centered at 4.7 Å^−1^, 1.52 Å) is higher than the equivalent peak in Co_H_Cu/Al_2_O_3_ (centered at 4.5 Å^−1^, 1.46 Å). This difference further confirms that Co_B_Cu/Al_2_O_3_ has longer Cu-O bonds, as the first shell is assigned to oxygen atoms coordinated to the copper.

**Fig. 2:**
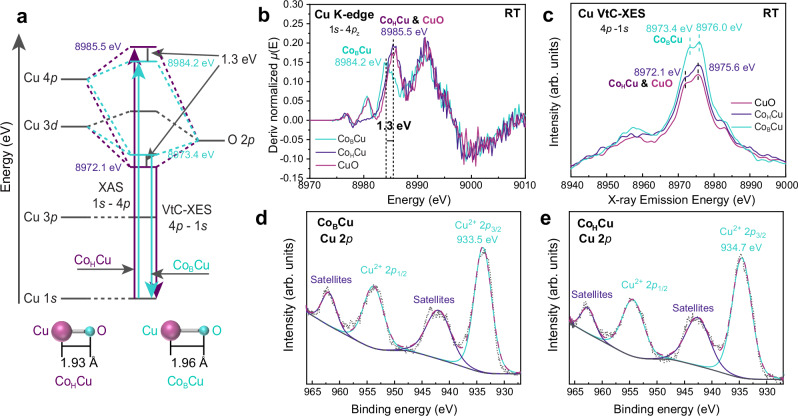
Characterization of Co_B_Cu/Al_2_O_3_ and Co_H_Cu/Al_2_O_3_. **a** simplified molecular orbital diagram of the CuO NPs in Co_B_Cu/Al_2_O_3_ and Co_H_Cu/Al_2_O_3_; **b** first derivative XANES spectra and **c** VtC-XES of Co_B_Cu/Al_2_O_3_ Co_H_Cu/Al_2_O_3_, and CuO/Al_2_O_3_; Cu 2*p* XPS spectra of Co_B_Cu/Al_2_O_3_ (**d**) and Co_H_Cu/Al_2_O_3_ (**e**).

The electronic structures of Co_B_Cu/Al_2_O_3_ and Co_H_Cu/Al_2_O_3_ were established using HERFD-XANES, valence-to-core X-ray emission spectroscopy (VtC-XES) and fine-scanned X-ray photoelectron spectroscopy (XPS) measurements (Figs. 2, S7). First derivative XANES contains a 1*s* → 4*p*_z_ transition at 8984.2 eV for Co_B_Cu/Al_2_O_3_ (Fig. 2b), which is 1.3 eV lower than in Co_H_Cu/Al_2_O_3_ and CuO/Al_2_O_3_. In previous studies^16,44^, the absorption energy for the 1*s* → 4*p*_z_ transition was shown to increase as the CuO loading decreases (from NPs to atomic sites), whereas a decrease in the 1 *s* → 4*p*_z_ transition energy is rarely observed. This discrepancy suggests a different local coordination environment of Cu^2+^ in Co_B_Cu/Al_2_O_3_ (Fig. 2b). Furthermore, VtC-XES reveals that the CuO NPs in Co_B_Cu/Al_2_O_3_ have a 1.3 eV higher Cu K_β2,5_ feature (mainly 4*p*→1*s* transitions) than the CuO NPs in Co_H_Cu/Al_2_O_3_ and CuO/Al_2_O_3_ (Fig. 2c). The distribution of Cu 4*p* states is markedly influenced by the hybridization between Cu 3 d and ligand p states^45^. For Cu^2+^ species, the low-energy peak observed in the main K_β2,5_ line primarily arises from the contribution of the π-bonding state^45^, which results from the hybridization of Cu 3*d*, O 2*p* and Cu 4*p* orbitals. The lower 1*s*→4*p*_z_ transition energy and higher π-bonding state of Co_B_Cu/Al_2_O_3_ point to different occupied and unoccupied states (Fig. 2a). Additionally, XPS of Co_B_Cu/Al_2_O_3_, Co_H_Cu/Al_2_O_3_, CuO/Al_2_O_3_, and CoCu/Al_2_O_3_ exhibit Cu^2+^ 2*p*_3/2_ peaks at 933.5, 934.7, 934.6, and 934.7 eV, respectively (Figs. 2d, e, S7c, d). Thus, the electronic states of Cu in Cu_H_Co, CuO/Al_2_O_3_, and CoCu/Al_2_O_3_ are similar, with that of Cu_B_Co being lower (Fig. S7c). The combination of HERFD-XANES, VtC-XES, and XPS suggests that the Cu-O bonds in the Co_B_Cu/Al_2_O_3_ catalyst are weakened relative to those observed in Co_H_Cu/Al_2_O_3_ (Fig. 2a), which is as expected, as the Cu-O bonds in Co_B_Cu/Al_2_O_3_ are longer (Fig. 1g, h). This feature indicates that the Cu-O bonds in Co_B_Cu/Al_2_O_3_ will be more reactive toward NH_3_, leading to the reduction of Cu^2+^ and concomitant oxidation of NH_3_. As proven in our recent publication, such a reaction is the rate-limiting step in the i-SCR mechanism^46^. Literature further supports this by showing that metal oxides with high metal–oxygen bond strengths exhibit lower rates in the NH_3_-SCO reaction^47^.

### Evaluation of the catalysts in the NH_3_-SCO reaction

**Fig. 3:**
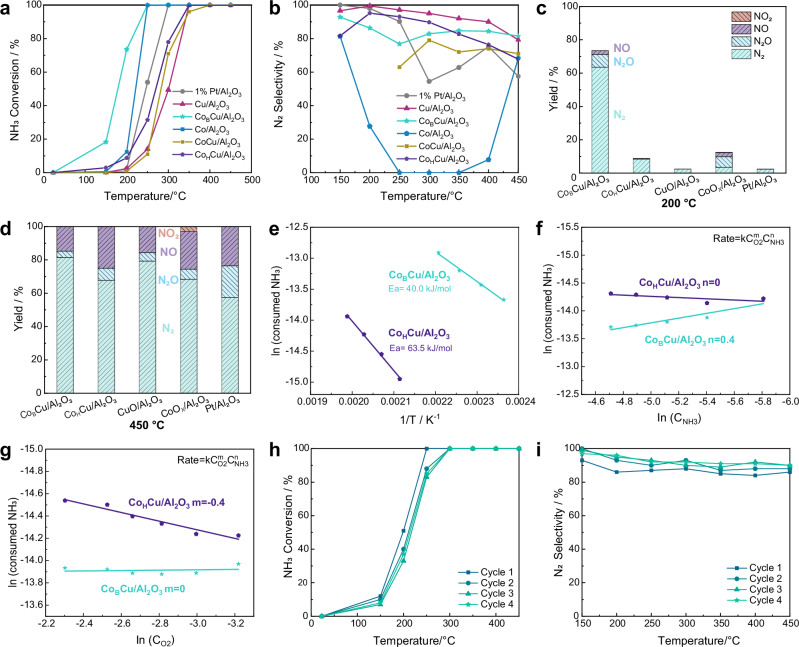
Evaluation of Co_B_Cu/Al_2_O_3_, Co_H_Cu/Al_2_O_3_ and control catalysts in the NH_3_-SCO reaction. NH_3_ conversion (**a**) and selectivity to N_2_ (**b**) as a function of temperature (note that CoCu/Al_2_O_3_ corresponds to the catalyst without reduction); Product distribution at 200 °C (**c**) and 450 °C (**d**); **e** Activation energy for the Co_B_Cu/Al_2_O_3_ and Co_H_Cu/Al_2_O_3_ catalyzed reaction; Reaction order for NH_3_ (**f**) and O_2_ (**g**); Stability tests of Co_B_Cu/Al_2_O_3_ over 4 cycles (**h**, **i**) (50 mg Co_B_Cu/Al_2_O_3_ mixed with 100 SiC). Reaction conditions: 50 mg catalyst, 5000 ppm NH_3_, 5% O_2_ balanced in He, gas flow: 100 mL/min, WHSV = 600 mL NH_3_·h^−^^1^·g^−1^.

The catalytic performance of the bimetallic Co_B_Cu/Al_2_O_3_ and Co_H_Cu/Al_2_O_3_ catalysts was compared with single metal CuO/Al_2_O_3_ and CoO_x_/Al_2_O_3_ catalysts in the NH_3_-SCO reaction (Figs. 3a, S12 and S13). 5000 ppm NH_3_ and GHSV 100,000 h^−^^1^ are used as the standard test conditions, which are consistent with waste gas streams in industrial processes and have been used in previous studies^48–51^.

Among these catalysts, Co_B_Cu/Al_2_O_3_ exhibits the highest activity with the lowest T_50_ value (i.e., the temperature that achieves 50% NH_3_ conversion) of around 175 °C, with complete NH_3_ conversion achieved at around 250 °C. The activities of Co_B_Cu/Al_2_O_3_ and Co_H_Cu/Al_2_O_3_ are higher than that of CoCu/Al_2_O_3_ (i.e., without reduction) (Fig. 3). Hence, both the addition of Co into CuO and the nature of the reductant affect the catalytic activity. Both Co_B_Cu/Al_2_O_3_ and Co_H_Cu/Al_2_O_3_ have higher activities than CuO/Al_2_O_3_, indicating that Co contributes to the activity (Fig. 3). Although CoO_x_/Al_2_O_3_ (5 wt% Co loading) is more active than Co_H_Cu/Al_2_O_3_, the selectivity to N_2_ is much lower, with the main products comprising NO and N_2_O between 250–350 °C (Fig. S10). At 200 °C, Co_B_Cu/Al_2_O_3_ showed at least a four-fold higher activity than the other catalysts (Fig. 3c). The Co loading was evaluated in the range of 0.1 to 5% for Co_B_Cu/Al_2_O_3_. As the Co loading increases, the Co species no longer remain as single sites, and the activity of Co_1%_Cu/Al_2_O_3_ (Co loading of 1 wt%) and Co_5%_Cu/Al_2_O_3_ (Co loading of 5 wt%) are not as high as Co_B_Cu/Al_2_O_3_ with a Co loading of 0.1 wt% (Fig. S14). Additionally, the selectivity to N_2_ for Co_B_Cu/Al_2_O_3_ was > 80%, even at high temperatures, and is lower than Co_H_Cu/Al_2_O_3_ below 350 °C, but is superior to Co_H_Cu/Al_2_O_3_ above 350 °C (Fig. 3b, d). Remarkably, despite having the same chemical composition, Co_B_Cu/Al_2_O_3_ displayed consistently higher activity than Co_H_Cu/Al_2_O_3_ across all temperature ranges. At 200 °C, Co_B_Cu/Al_2_O_3_ achieved nearly 10 times higher NH_3_ conversion than Co_H_Cu/Al_2_O_3_ (Fig. 3a). The different electronic structures lead to differences in the strength of the metal–oxygen bonds, which influence both activity and selectivity in the NH_3_-SCO reaction^38,39^. Catalysts with weaker metal–oxygen bonds tend to exhibit higher rates of reaction, but result in the formation of NO_x_ (NO and N_2_O). Compared to Co_H_Cu/Al_2_O_3_, Co_B_Cu/Al_2_O_3_ has a lower 1*s* → 4*p*_z_ antibonding transition and a higher 4*p* →1*s* bonding transition of Cu-O bonds, indicative of weaker Cu–O bonds^52–54^. Thus, the superior activity and lower selectivity to N_2_ observed for Co_B_Cu/Al_2_O_3_ may be attributed to the electronic structure of Cu^2+^ produced by the local coordination environment.

The apparent activation energy of Co_B_Cu/Al_2_O_3_ is 40.0 kJ/mol, which is lower than that of Co_H_Cu/Al_2_O_3_ with a value of 63.5 kJ/mol (Fig. 3e). This difference suggests that the superior activity of Co_B_Cu/Al_2_O_3_ in the NH_3_-SCO reaction may be attributed to a reduced energy barrier for the reaction. The reaction order for NH_3_ in the SCO process is 0.4 for Co_B_Cu/Al_2_O_3_, indicating partial dependence on NH_3_ concentration, while it is 0 for Co_H_Cu/Al_2_O_3_ (Fig. 3f), implying that NH_3_ does not influence the reaction rate under these conditions due to a much higher Cu-O bond energy. The reaction order for O_2_ also differs between the catalysts, with Co_B_Cu/Al_2_O_3_ exhibiting an O_2_ order of 0 (Fig. 3g), which suggests the participation of lattice oxygen in the reaction. In contrast, Co_H_Cu/Al_2_O_3_ shows an O_2_ order of −0.4 (Fig. 3g), likely due to competitive adsorption between NH_3_ and O_2_ at active sites, which inhibits O_2_ involvement in the reaction. Furthermore, Co_B_Cu/Al_2_O_3_ demonstrates good stability under the reaction conditions, showing no significant decrease in catalytic activity or N_2_ selectivity even after four consecutive reaction cycles. The reduced activity might be caused by the weak metal-support interactions between nanoparticles and support.

### Redox properties of the CuO NPs

The redox properties of the catalyst play a crucial role in the oxidation of NH_3_, directly impacting on the overall NH_3_ oxidation activity^46^. The performance of Cu-based catalysts is related to the Cu^+^/Cu^2+^ redox couple, and the activity may be correlated with the presence of Cu^+^, as observed in several Cu-based catalysts^55,56^. Mechanistically, the reaction could be considered to proceed through the reaction of NH_3_ with Cu^2+^ to form Cu^I^(NH_3_)_2_ as the rate-determining step, followed by the oxidation of Cu^I^(NH_3_)_2_ to Cu^2+^.

**Fig. 4:**
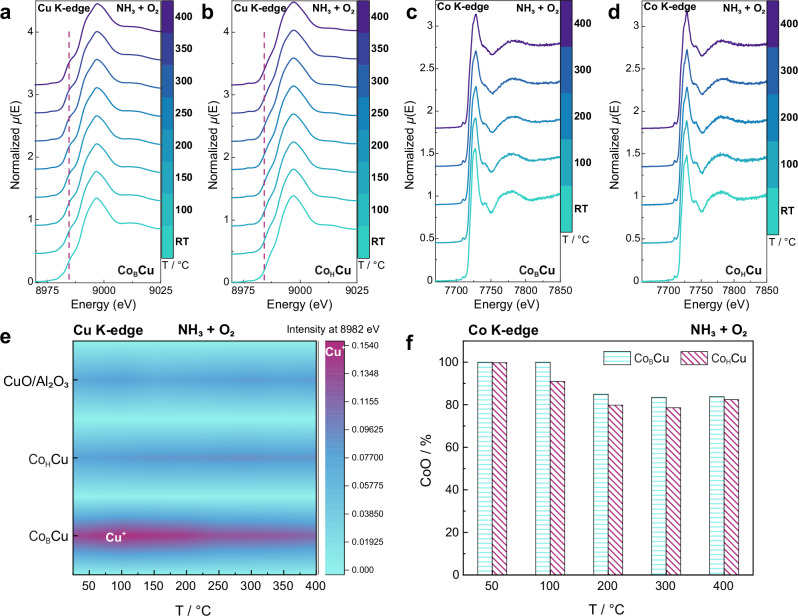
Operando studies of the redox behavior of Cu and Co in Co_B_Cu/Al_2_O_3_ and Co_H_Cu/Al_2_O_3_. *Operando* Cu K-edge XAFS of Co_B_Cu/Al_2_O_3_ (**a**) and Co_H_Cu/Al_2_O_3_ (**b**) as a function of temperature; *operando* Co K-edge XAFS of Co_B_Cu/Al_2_O_3_ (**c**) and Co_H_Cu/Al_2_O_3_ (**d**); **e** *operando* Cu K-edge XAFS, signal intensity of the Cu^+^ 1 *s* → 4*p* transition peak at 8982 eV in a NH_3_/O_2_ atmosphere as a function of temperature; **f** proportion of CoO in Co_B_Cu/Al_2_O_3_ and Co_H_Cu/Al_2_O_3_ as a function of temperature.

To evaluate the redox behavior of the single Co and bulk Cu sites, and monitor structural changes under real NH_3_-SCO reaction conditions, *operando* XAFS was undertaken in fluorescence mode at the Co K-edge and transmission mode at the Cu K-edge under steady-state conditions at each temperature (Figs. 4, S15 and S16). The formation of reduced Cu^+^ is considered to be a trigger for the NH_3_-SCO reaction. In the Cu K-edge XAFS spectra, the evolution of the Cu^+^ 1*s* → 4*p* transition peak of Co_B_Cu/Al_2_O_3_, Co_H_Cu/Al_2_O_3_ and CuO/Al_2_O_3_ was monitored as a function of temperature during the NH_3_-SCO reaction (NH_3_ 5000 ppm, O_2_ 5%) (Fig. 4e). The Cu^2+^ in Co_B_Cu/Al_2_O_3_ is partially reduced to Cu^+^, even under excess O_2_, as evidenced by the more pronounced peak intensity at Cu K-edge 8982 eV (the typical feature for Cu^I^(NH_3_)_2_ 1*s* → 4*p*, XANES) (Fig. 4e). In contrast, the peak intensity at Cu K-edge 8982 eV in Co_H_Cu/Al_2_O_3_ slightly increases, and Cu^+^ is not detected throughout the entire temperature range in CuO/Al_2_O_3_ (Fig. 4e, S19 and 20). The redox activity of the single Co sites in Co_B_Cu/Al_2_O_3_ and Co_H_Cu/Al_2_O_3_ is similar (Fig. 4f). The single Co sites comprise Co^2+^ at room temperature and are partially oxidized to Co_3_O_4_ as the temperature increases. Moreover, in different gases from the most reductive, i.e., NH_3_ without O_2_, to the most oxidative, i.e., NH_3_ + O_2_, the Cu species in Co_B_Cu/Al_2_O_3_ are in a more reduced state than Co_H_Cu/Al_2_O_3_ (Figs. S17–19), confirming the superior redox ability of the CuO NPs in Co_B_Cu/Al_2_O_3_.

**Fig. 5:**
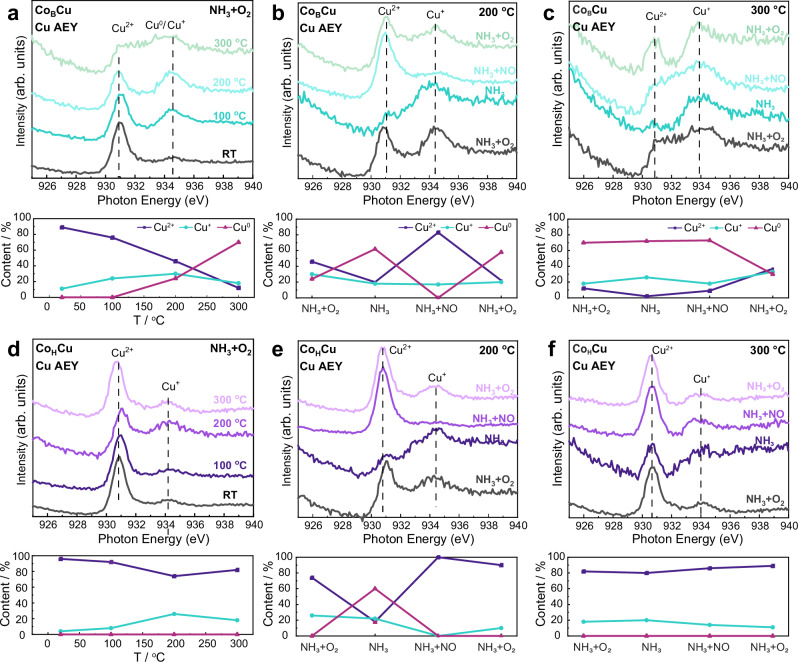
In-situ NAP-NEXAFS spectra and corresponding Cu species distribution of Co_B_Cu/Al_2_O_3_ and Co_H_Cu/Al_2_O_3_. **a** Cu L-edge (in Auger electron yield (AEY) mode) and corresponding Cu species content of Co_B_Cu/Al_2_O_3_ under NH_3_ + O_2_ as a function of temperature; Cu L-edge and corresponding Cu species content of Co_B_Cu/Al_2_O_3_ under various gas atmospheres at 200 °C (**b**) and at 300 °C (**c**) (gas pressure 0.3 mbar); **d** Cu L-edge (AEY mode) and corresponding Cu species content of Co_H_Cu/Al_2_O_3_ under NH_3_ + O_2_ as a function of temperature; Cu L-edge (AEY mode) and corresponding Cu species content of Co_H_Cu/Al_2_O_3_ under various gas atmospheres at 200 °C (**e**) and at 300 °C (**f**) (gas pressure 0.3 mbar).

As the catalytically active sites are predominantly on the surface, in-situ near ambient pressure near-edge X-ray absorption fine structure (NAP-NEXAFS) was used to investigate the redox properties of the surface Cu species in Co_B_Cu/Al_2_O_3_ and Co_H_Cu/Al_2_O_3_ (Fig. 5). The redox activity of the Cu species in Co_B_Cu/Al_2_O_3_ and Co_H_Cu/Al_2_O_3_ was evaluated under NH_3_ + O_2_ at different temperatures (Fig. 5a, d). At all temperatures, Co_B_Cu/Al_2_O_3_ has a more pronounced peak compared to Co_H_Cu/Al_2_O_3_ at 934.2 eV corresponding to Cu^+^/Cu^0^, and a broad peak at 930.9 eV corresponding to Cu^2+^. Linear combination fitting (LCF) (see standards in Fig. S20) further confirms that the proportion of reductive Cu species (Cu^+^/Cu^0^) in Co_B_Cu/Al_2_O_3_ is higher than in Co_H_Cu/Al_2_O_3_ at all temperatures. Above 200 °C, the reduced Cu species dominate in Co_B_Cu/Al_2_O_3_ catalyst.

Additionally, NAP-NEXAFS of the Co_B_Cu/Al_2_O_3_ and Co_H_Cu/Al_2_O_3_ catalysts under different gas atmospheres further confirmed the superior redox properties of the single Cu sites in Co_B_Cu/Al_2_O_3_ (Fig. 5). At 200 °C in NH_3_, the surface Cu species of both Co_B_Cu/Al_2_O_3_ and Co_H_Cu/Al_2_O_3_ are nearly completely reduced to Cu^+^/Cu^0^. At 300 °C in NH_3_, the surface Cu species of Co_B_Cu/Al_2_O_3_ are nearly completely reduced to Cu^+^/Cu^0^, whereas the surface Cu species of Co_H_Cu/Al_2_O_3_ are mainly in the form of Cu^2+^. Under NH_3_ + NO, the surface Cu species in Co_H_Cu/Al_2_O_3_ are more oxidized than the surface Cu species in Co_B_Cu/Al_2_O_3_, which become more reduced. Based on the *operando* XAFS and in-situ NAP-NEXAFS studies, the Cu species in the CuO NPs with weaker Cu-O bonds, as in Co_B_Cu/Al_2_O_3_, exhibit superior redox properties. The longer (and hence weaker) Cu-O bonds in Co_B_Cu/Al_2_O_3_ result in enhanced redox ability and, consequently, higher activity.

**Fig. 6:**
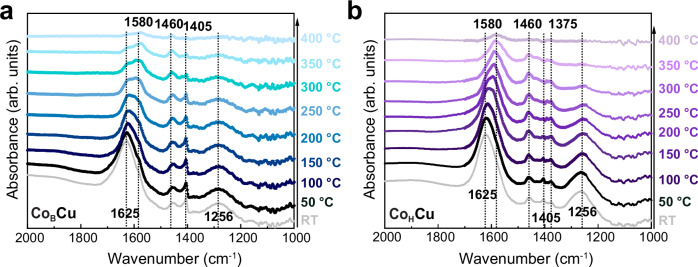
In-Situ DRIFTS spectra. In-Situ DRIFTS spectra of Co_B_Cu/Al_2_O_3_ (**a**) and Cu_H_Cu/Al_2_O_3_ (**b**) as a function of temperature (the catalysts were exposed to a flow of 5000 ppm NH_3_ and 5% O_2_ for 20 min at different temperatures.

In-Situ DRIFTS confirms different adsorption behavior in Co_B_Cu/Al_2_O_3_ and Co_H_Cu/Al_2_O_3_ (Fig. 6)^57^. The bands observed at 1625 and 1256 cm^−1^ may be assigned to asymmetric and symmetric deformation of ammonia chemisorbed on Lewis acid sites of Al_2_O_3_, respectively^23^. The peak at 1405 cm^−1^ may be assigned to NH_3_ coordinated to Cu^58,59^. The peak at 1460 cm^−1^ originates from Brønsted acid sites on Al_2_O_3_. A peak at 1580 cm^–1^ gradually emerges at temperatures above 250 °C in Co_B_Cu/Al_2_O_3_, which may be tentatively assigned to nitrate species, with this peak only emerging at temperatures above 300 °C in the presence of Co_B_Cu/Al_2_O_3_. The unique electronic structure of the CuO NPs in Co_B_Cu/Al_2_O_3_ which leads to weaker Cu-O bonds, might be an important factor for the enhanced redox activity and the distinct adsorption behavior, which leads to a better catalytic performance, compared to Co_H_Cu/Al_2_O_3_.

NH_3_ emissions are expected to rise in the future from mobile vehicles and other industries, as there is increasing interest in using NH_3_ as a sustainable fuel in particularly for shipping. Consequently, NH_3_-SCO to N_2_ is a promising approach to help mitigate NH_3_ emissions. To improve the performance of earth-abundant metal CoCu-based bimetallic catalysts, single Co sites were doped onto CuO NPs immobilized on an Al_2_O_3_ support, affording Co_B_Cu/Al_2_O_3_ and Co_H_Cu/Al_2_O_3_ catalysts with different electronic structures. Notably, the Co_B_Cu/Al_2_O_3_ catalyst outperforms the Co_H_Cu/Al_2_O_3_ catalysts with respect to both activity and selectivity, achieving full conversion of 5000 ppm NH_3_ at 250 °C, with a selectivity to N_2_ > 80%, which is superior to the commercial 1% Pt/Al_2_O_3_ catalyst. The difference in the catalytic performance may be traced to differences in the electronic structure of the CuO NPs in Co_B_Cu/Al_2_O_3_ and Co_H_Cu/Al_2_O_3_ using advanced HERFD-XANES and VtC-XES techniques. The lower 1*s* → 4*p*_z_ transition and higher 1*s* → 4*p* transition (K_β2,5_ feature) of CuO in Co_B_Cu/Al_2_O_3_ correlate with weaker Cu-O bonds, and these weaker bonds are related to longer Cu-O bonds lengths, as confirmed by STEM, EXAFS and wt-EXAFS. The weaker Cu-O bonds lead to enhanced redox properties of the CuO NPs, and enhanced NH_3_-SCO activity, further validated by *operando* XAS and in-situ NAP-NEXAFS studies. In contrast to the other catalysts, the oxidation state of the Cu center in Co_B_Cu/Al_2_O_3_ is able to switch between Cu^2+^ and Cu^+^ under all gas conditions and at all temperatures studied, which is vital for the catalyst to maintain good performance under real world conditions. Future studies will focus on upscaling and demonstrating the Co_B_Cu/Al_2_O_3_ catalyst with real fuel gases.

## Methods

### Materials

Cu(NO_3_)_2_^.^3H_2_O, Co(NO_3_)_2_^.^6H_2_O, and NaBH_4_ were purchased from Sigma Aldrich, and γ-Al_2_O_3_ was from Johnson Matthey. All chemicals are used as received.

### Catalyst preparation

#### Synthesis of Co_B_Cu/Al_2_O_3_

The Co_B_Cu/Al_2_O_3_ catalyst was prepared via co-reduction of Co and Cu precursors using NaBH_4_ as the reducing agent, followed by natural oxidation in the air. γ-Al_2_O_3_ (0.5 g, Johnson Matthey) was dispersed in H_2_O (10 mL) under sonication for 30 min. Solution A containing Cu(NO_3_)_2_^.^3H_2_O (100 mg) in H_2_O (2 mL) and Solution B containing Co(NO_3_)_2_^.^6H_2_O (2.5 mg) in H_2_O (1 mL), were slowly added to the Al_2_O_3_ suspension, avoiding contact with air. After cooling in the ice bath, NaBH_4_ (78 mg) in H_2_O (2 mL) was added, and the resulting suspension was stirred for 30 min under N_2_. The resulting solid was collected by centrifugation and washed with H_2_O (5 ×30 mL), and dried under vacuum, then followed by natural oxidation in the air to afford Co_B_Cu/Al_2_O_3_.

#### Synthesis of Co_H_Cu/Al_2_O_3_

The Co_H_Cu/Al_2_O_3_ catalyst was prepared via co-reduction of Co and Cu precursors under H_2_, followed by natural oxidation in the air. γ-Al_2_O_3_ (0.5 g, Johnson Matthey) was dispersed in H_2_O (10 mL) under sonication for 30 min. Solution A containing Cu(NO_3_)_2_^.^3H_2_O (100 mg) in H_2_O (2 mL) and Solution B containing Co(NO_3_)_2_^.^6H_2_O (2.5 mg) in H_2_O (1 mL), were slowly added to the Al_2_O_3_ suspension. The resulting suspension was heated at 60 °C under stirring until all the solvent had evaporated. The remaining solid was heated to 300 °C for 1 h under 15% H_2_/Ar at a heating rate of 5 °C/min in a tube furnace, then followed by natural oxidation in the air to afford Co_H_Cu/Al_2_O_3_.

#### Synthesis of CuO/Al_2_O_3_

γ-Al_2_O_3_ (0.5 g, Johnson Matthey) was dispersed in H_2_O (10 mL) with vigorous stirring at room temperature. To the resulting suspension, a solution containing Cu(NO_3_)_2_^.^3H_2_O (100 mg) in H_2_O (2 mL) was slowly added. The reaction mixture was then heated at 60 °C under stirring until all the solvent had evaporated. The remaining solid was heated to 300 °C for 1 h under 15% H_2_/Ar at a heating rate of 5 °C/min in a tube furnace, then followed by natural oxidation in the air to afford CuO/Al_2_O_3_.

#### Synthesis of CoO_x_/Al_2_O_3_

γ-Al_2_O_3_ (0.5 g, Johnson Matthey) was dispersed in H_2_O (10 mL) under sonication for 30 min. Solution containing Co(NO_3_)_2_^.^6H_2_O (130 mg) in H_2_O (2 mL) was slowly added to the Al_2_O_3_ suspension. The resulting suspension was heated at 60 °C under stirring until all the solvent had evaporated. The remaining solid was heated to 300 °C for 1 h under 15% H_2_/Ar at a heating rate of 5 °C/min in a tube furnace, then followed by natural oxidation in the air to afford CoO_x_/Al_2_O_3_.

#### Synthesis of CoCu/Al_2_O_3_ (i.e., without reduction)

γ-Al_2_O_3_ (0.5 g, Johnson Matthey) was dispersed in H_2_O (10 mL) under sonication for 30 min. Solution A containing Cu(NO_3_)_2_^.^3H_2_O (100 mg) in H_2_O (2 mL) and Solution B containing Co(NO_3_)_2_^.^6H_2_O (2.5 mg) in H_2_O (1 mL), were slowly added to the Al_2_O_3_ suspension. The resulting suspension was heated at 60 °C under stirring until all the solvent had evaporated. The remaining solid is CoCu/Al_2_O_3_.

### Ex situ characterization

X-ray diffraction (XRD) patterns were recorded using a StadiP diffractometer (STOE) equipped with a Mo source (Kα = 0.7093165 Å). The instrument operated at an applied voltage of 40 kV and a current of 30 mA. Diffraction data in the 2θ range of 2–40° were systematically collected, with a resolution of 0.015° per step.

X-ray photoelectron spectroscopy (XPS) was conducted using a Thermo Fisher Scientific NEXSA spectrometer. The C 1*s* peak at 284.8 eV served as the standard reference for calibrating the photoelectron energy shift. CasaXPS software was used for data analysis.

Energy dispersive X-ray (EDX) spectroscopy and aberration-corrected bright field (BF) imaging were performed on a JEOL ARM200CF (E01) operating at 200 kV and equipped with JEOL dual silicon drift detectors at the electron Physical Sciences Imaging Centre (ePSIC) at the Diamond Light Source (DLS) (UK). The instrument operated with a convergence semi-angle of 23.0 mrad with BF collection semi-angles of 0–21.9. Single-pass EDX spectra were collected with drift correction. Data were acquired and processed using the Gatan Microscopy Suite (a.k.a. Digital Micrograph)^60^. Samples were prepared via a standard preparation route: a small amount (<20 mg) of catalyst powder was dispersed in approximately 5 ml of ethanol, after sonication and drop casting approximately 1 ml of supernatant onto holey carbon-coated, gold TEM support grids. Gold was used instead of the more typical copper grid to avoid overlapping fluorescent signals with the sample during EDX mapping. The average particle size was calculated based on more than 100 particles for each sample.

HERFD-XANES measurements were conducted at the I20-Scanning beamline at the DLS (UK). The X-ray beam was introduced through Rh-coated optic hutch mirrors, and a Si(111) scanning four-bounce monochromator was employed to select the incident energy. HERFD-XANES spectra were obtained by scanning the incident energy across the range 8800.00 to 9400.00 eV with a step size of 0.15 eV. Samples were homogenized with boron nitride and compressed into pellets with a diameter of 8 mm. XANES analysis was carried out using the Demeter software package.

Extended X-ray absorption fine structure (EXAFS) of the Co K-edge (7709 eV) and Cu K-edge (8979 eV) were carried out at the DLS (UK) and Spring8 (Japan). Samples were directly pressed into pellets for fluorescence measurements of the Co K-edge and transmission measurements of the Cu K-edge. Co foil or Cu foil standards were used for energy shift calibration. XAFS data were merged from 3 spectra to improve signal quality and were processed using the Demeter software package (including Athena and Artemis). Athena software was used to analyze the XANES data. Artemis software was used to fit the *k*^2^-weighted EXAFS data in real space with 3.0 Å^−1^ < *k* < 12.0 Å^−1^ and 1.0 Å <*R* < 3.3 Å. The calculated amplitude reduction factor, S_0_^2^, from the EXAFS analysis of Cu and Co foil was 0.878 and 0.879, respectively, and were used as fixed parameters for EXAFS fitting. The coordination number and bond length were calculated based on the reported structures from the Crystal open database: Cu (No.9013014), CuO (No. 1011148), Co (No. 9008492), and Co_3_O_4_ (No. 9005898).

### *Operando* Co K-edge and Cu K-edge XAFS

*Operando* XAFS experiments were performed at the Spring8 beamline (Japan). 100 mg of pelletized catalysts were measured at 8780–10200 eV for Cu K edge in transmission mode and 7505–8670 eV for Co K-edge in fluorescence mode at different temperatures and under various gas atmospheres. The pelletized catalyst was exposed to 5000 ppm NH_3_ and 5% O_2_ in He (total gas flow rate 100 mL/min) at different temperature from 30 °C to 450 °C. For experiments carried out under various gas atmospheres at 200 °C, the sequence of different gas atmospheres follows NH_3_ + O_2_ (5000 ppm NH_3_ and 5% O_2_ in He), NH_3_ (5000 ppm NH_3_ in He), NH_3_ + O_2_ (5000 ppm NH_3_ and 5% O_2_ in He), and O_2_ (5% O_2_ in He). All spectra were recorded under steady-state conditions. Spectra processing was performed with Athena software.

### In-situ DRIFTS

DRIFTS were performed on a PerkinElmer Frontier FT-IR Spectrometer. The sample was heated in He at 350 °C for 30 min to remove surface contamination. After cooling to room temperature, the sample was exposed to 5000 ppm NH_3_ and 5% O_2_ in He (total gas flow rate 100 mL/min), for 30 min, during which spectra were recorded. Then, the sample was heated from 30 °C to 450 °C with a temperature ramp of 10 °C/min. The spectra were recorded from 4400 to 500 cm^-1^ with a resolution of 2 cm^−1^. Background spectra were recorded in He and subtracted from the sample spectrum for each measurement.

### In situ near-edge X-ray absorption fine structure (NEXAFS) spectroscopy

In situ NEXAFS experiments were performed on the B07 beamline at the DLS (UK)^61^. The X-ray radiation was sourced from a bending magnet and a plane grating monochromator (PGM) with an energy range from 110 to 2800 eV (soft X-ray range) and flux of >10^10^ photons/s with 0.3 A ring current. The reaction products were monitored online using an electron impact mass spectrometer (Hiden HMT100) connected directly to the prelens chamber. The pressure in the specimen chamber was precisely controlled (HV or 0.1–1 mbar) by simultaneous operation of several mass flow controllers for reactive gases and a PID-controlled throttle valve acting as back pressure controller. Temperature control was provided by two K-type thermocouples. NEXAFS spectra at Cu L-edge (925–940 eV) were measured in Auger electron yield (AEY) mode using a SPECS phoibos 150 hemispherical analyser set.

### Catalytic performance measurements

The performance of the catalysts in the NH_3_-SCO reaction was evaluated in a fixed-bed flow reactor at a gas flow rate of 100 mL/min, which consists of 5000 ppm NH_3_, 5% O_2_, and He balance. Typically, 50 mg of catalyst was placed in the reaction tube, and the quantification of products was performed with an online quadrupole mass spectrometer quantitative gas analyzer (Hiden Analytical, UK). The reactions were investigated at temperatures ranging from 100 to 450 °C and kept stable for at least 30 min after attaining a steady state at each reaction temperature to detect the MS signals of NH_3_ and O_2_ and the products, i.e., N_2_, N_2_O, and NO. Stability test: 50 mg Co_B_Cu/Al_2_O_3_ and 100 mg SiC were mixed by grinding and then tested for 4 cycles.

## Supplementary information

Supplementary Information

Transparent Peer Review file

## Data Availability

All data generated in this study are provided in the Supplementary Information. Data are available from the corresponding authors upon request.
