# Supplementary material for: Lowering the Cu-O bond energy in CuO nanocatalysts enhances the efficiency of NH3 oxidation
Source: Nat Commun. 2025 Oct 24;16:9412. doi: 10.1038/s41467-025-64415-w (PMC12552456; doi:10.1038/s41467-025-64415-w)
Supplement: Supplementary file 1 — Supplementary Information [file 41467_2025_64415_MOESM1_ESM.pdf]

Supplementary Information

Lowering the Cu-O bond energy in CuO nanocatalysts enhances the efficiency of NH_3 oxidation

Lu Chen[1,2,3*], Xuze Guan[2], Zhangyi Yao[2], Shusaku Hayama[4], Matthijs A. van Spronsen[4], Burcu Karagoz[4], Georg Held[4], David G. Hopkinson[5], Christopher S. Allen[5,6], June Callison[7], Paul J. Dyson[8,*], Feng Ryan Wang[2,*]

1. Low-Carbon Conversion Science and Engineering Center, Shanghai Advanced Research Institute, Chinese Academy of Sciences, 201210, Shanghai, China
2. Department of Chemical Engineering, University College London, Roberts Building, Torrington Place, WC1E 7JE, London, United Kingdom
3. Yusuf Hamied Department of Chemistry, University of Cambridge, CB2 1EW, Cambridge, United Kingdom
4. Diamond Light Source Ltd., Harwell Science and Innovation Campus, Chilton, OX11 0DE, Didcot, United Kingdom
5. electron Physical Science Imaging Centre, Diamond Light Source Ltd., OX11 0DE, Didcot, United Kingdom
6. Department of Materials, University of Oxford, OX1 3PH, Oxford, United Kingdom
7. UK Catalysis Hub, Research Complex at Harwell (RCaH), Rutherford Appleton Laboratory, OX11 0FA, Harwell, United Kingdom
8. Institute of Chemical Sciences and Engineering, École Polytechnique Fedérale de Lausanne (EPFL), 1015, Lausanne, Switzerland

* e-mail: chenlu@sari.ac.cn, paul.dyson@epfl.ch, ryan.wang@ucl.ac.uk

This PDF file includes:

Figs. S1 to S20
Tables S1

Contents

Fig. S1. STEM images and particle size distribution of Co_BCu/Al_2O_3 before reaction. 3
Fig. S2. STEM images and particle size distribution of Co_HCu/Al_2O_3 before reaction. 4
Fig. S3. STEM images and particle size distribution of Co_BCu/Al_2O_3 after reaction. 5
Fig. S4. STEM images and particle size distribution of Co_HCu/Al_2O_3 after reaction.. 6
Fig. S5. Compositional elemental mapping of Co_BCu/Al_2O_3. 7
Fig. S6. Electron paramagnetic resonance (EPR) spectra. 8
Fig. S7. X-Ray diffraction (XRD) patterns and XPS spectra. 9
Fig. S8. Hydrogen temperature-programmed reduction (H_2-TPR). 10
Fig. S9. EXAFS fitting of Cu foil and Co foil.. 11
Fig. S10. EXAFS fitting of Co_BCu/Al_2O_3. 12
Fig. S11. EXAFS fitting of Co_HCu/Al_2O_3. 13
Fig. S12. Performance of the catalysts in the oxidation of NH_3 at different temperatures. a Co_BCu/Al_2O_3 and b Co_HCu/Al_2O_3. 14
Fig. S13. Performance of the catalysts in the oxidation of NH_3 at different temperatures........... 15
Fig. S14. Performance of Co_BCu/Al_2O_3 with different Co loading in the oxidation of NH_3 at different temperatures. 16
Fig. S15. *Operando* Cu K-edge EXAFS spectra at different reaction temperatures. 17
Fig. S16. *Operando* Co K-edge EXAFS spectra at different reaction temperatures. 18
Fig. S17. *Operando* Cu K-edge XANES spectra of different catalysts under different gases. 19
Fig. S18. *Operando* Cu K-edge EXAFS spectra in different gases 20
Fig. S19. *Operando* Co K-edge EXAFS spectra in different gases. 21
Fig. S20. Cu L-edge (in Auger electron yield (AEY) mode) of Cu foil, Cu_2O and CuO. 22
Table S1. EXAFS fitting results. 23

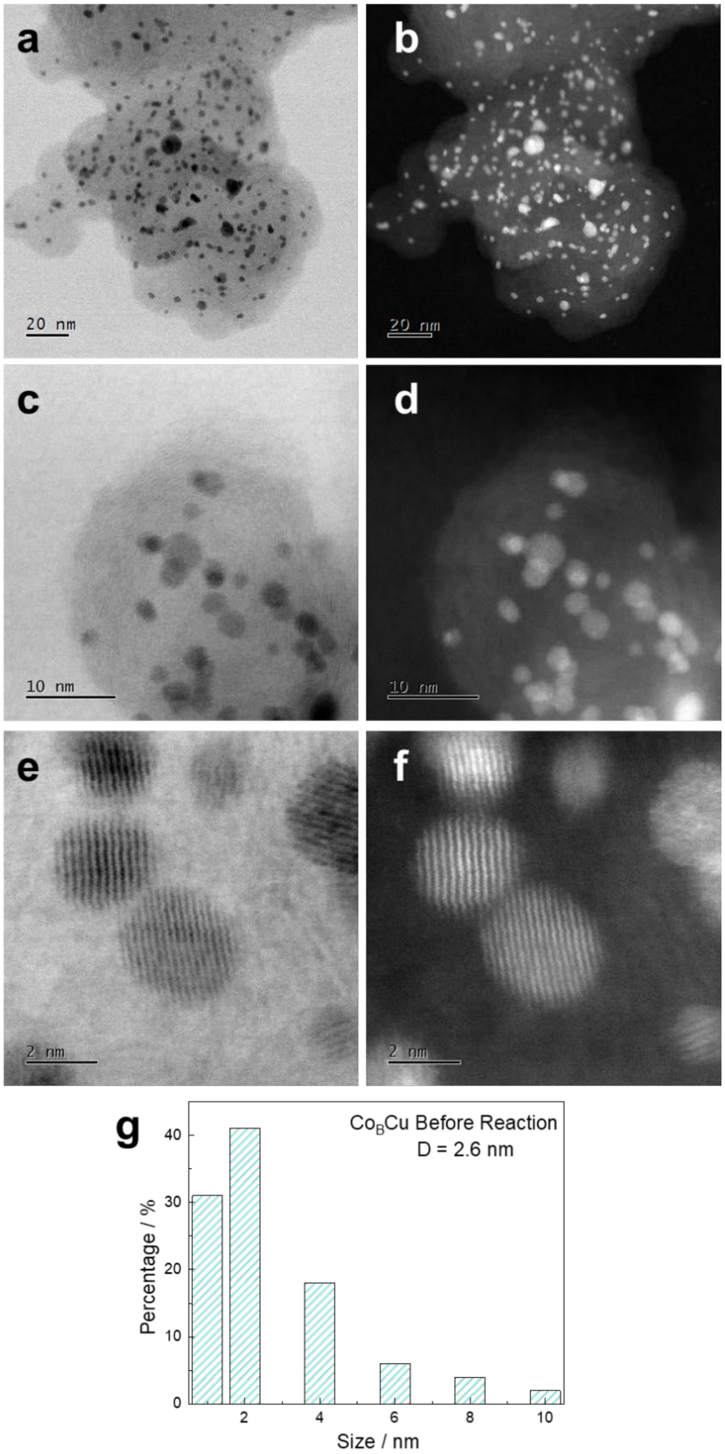

Fig. S1. STEM images and particle size distribution of Co_BCu/Al_2O_3 before reaction. BF-STEM (**a, c, e**) and HAADF-STEM **(b, d, f**) images of Co_BCu/Al_2O_3 before reaction at different magnifications and the particle size distribution (**g**). The average particle size was calculated based on more than 100 particles.

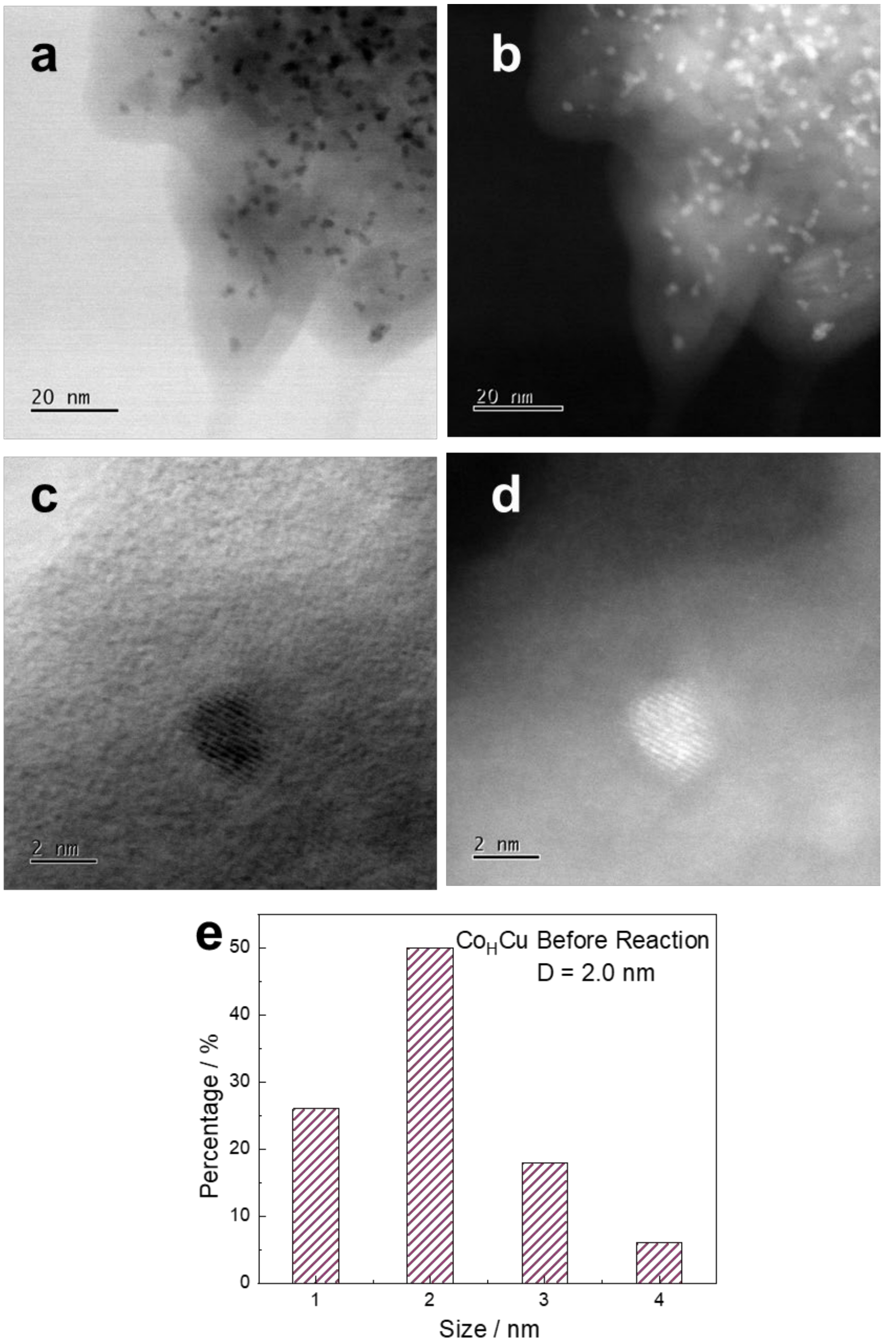

Fig. S2. STEM images and particle size distribution of Co_HCu/Al_2O_3 before reaction. BF-STEM (**a, c**) and HAADF-STEM (**b, d**) images of Co_BCu/Al_2O_3 before reaction at different magnifications and the particle size distribution (**e**). The average particle size was calculated based on more than 100 particles.

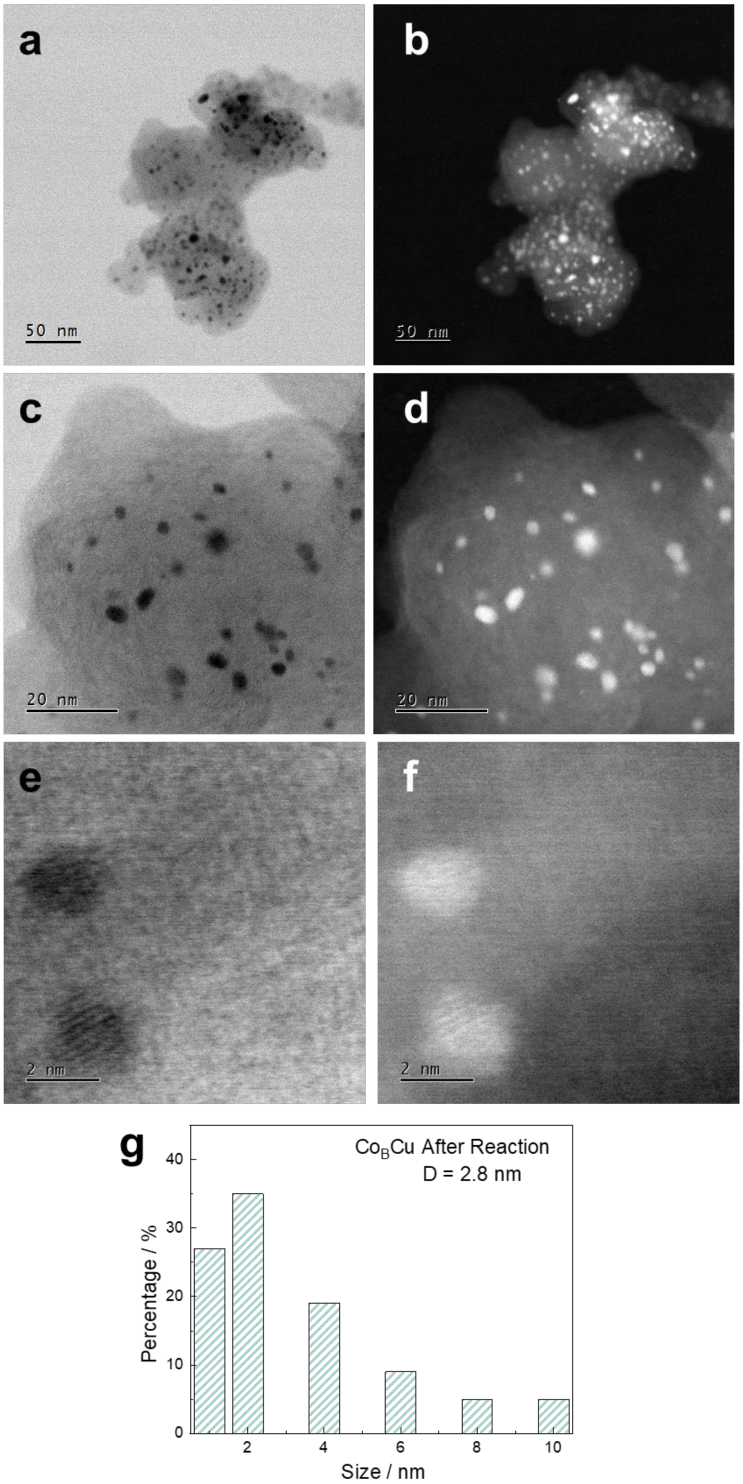

Fig. S3. STEM images and particle size distribution of Co$_B$Cu/Al_2O_3 after reaction. BF-STEM (**a, c, e**) and HAADF-STEM **(b, d, f)** images of Co$_B$Cu/Al_2O_3 before reaction at different magnifications and the particle size distribution (**g**).The average particle size was calculated based on more than 100 particles.

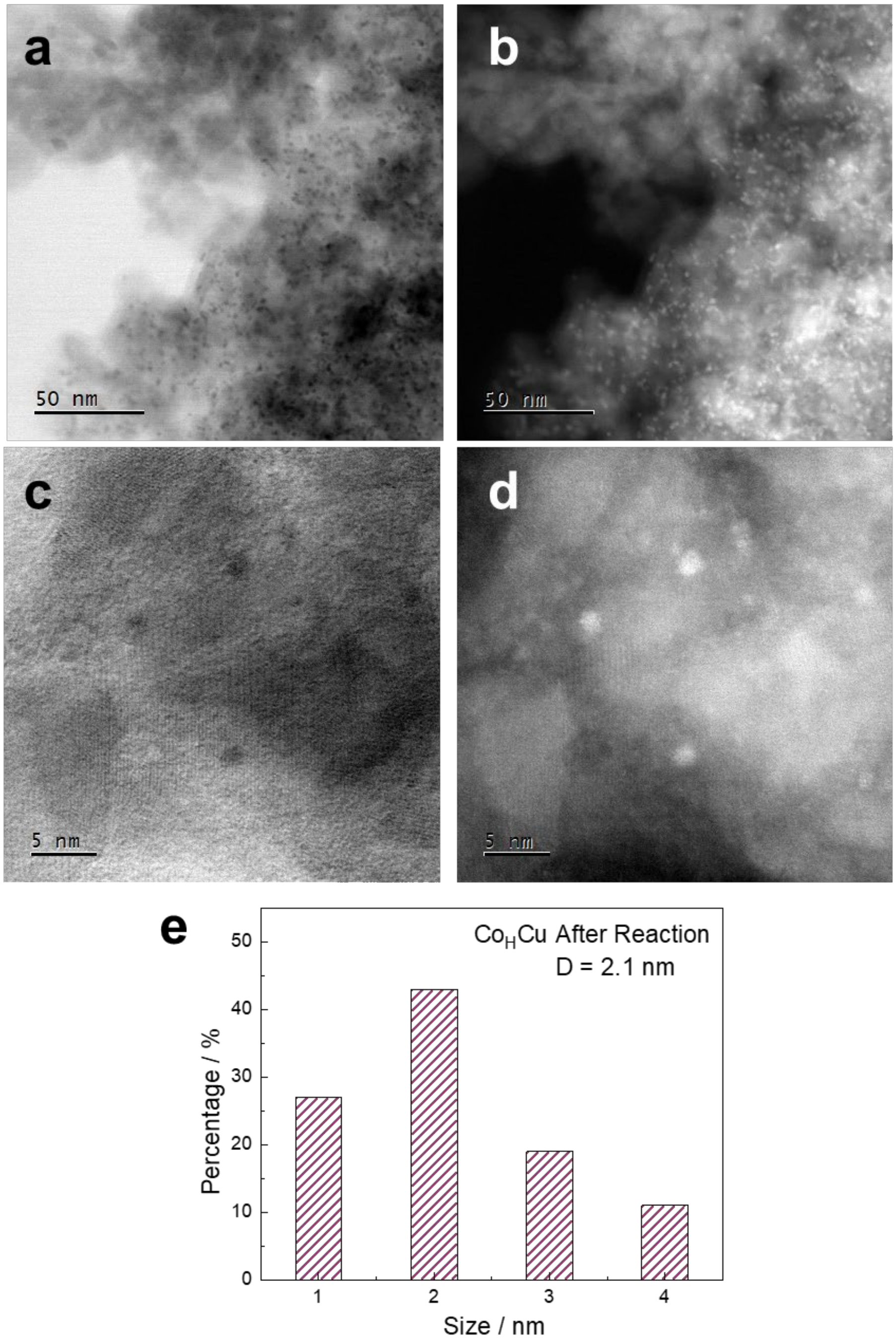

Fig. S4. STEM images and particle size distribution of Co_HCu/Al_2O_3 after reaction. BF-STEM (**a, c**) and HAADF-STEM **(b, d**) images of Co_BCu/Al_2O_3 before reaction at different magnifications and the particle size distribution (**e**). The average particle size was calculated based on more than 100 particles.

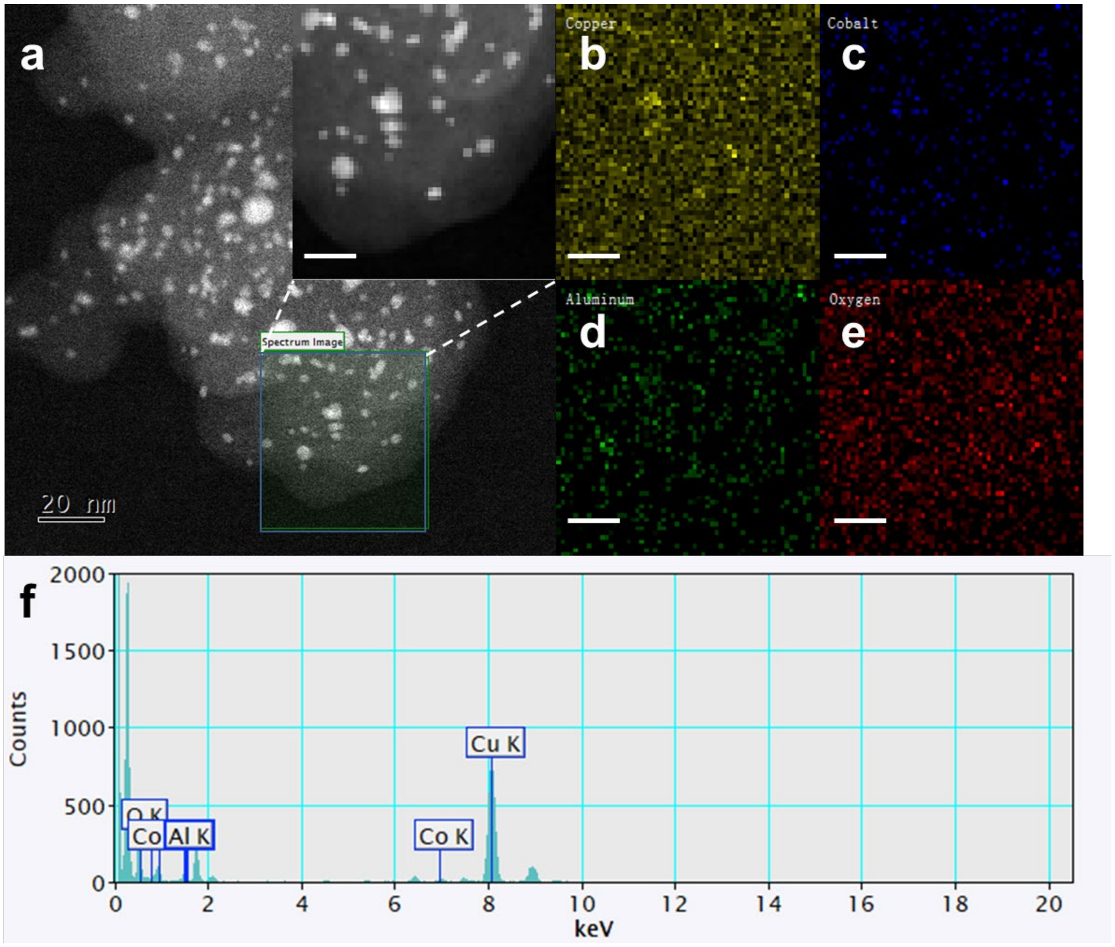

Fig. S5. Compositional elemental mapping of Co_BCu/Al_2O_3. a STEM image of Co_BCu/Al_2O_3 (inserted image: EDS mapping area, scale bar: 10 nm); **b-e** EDS elemental mapping of Co_BCu/Al_2O_3 (scale bar: 10 nm); **f** EDX element distribution pattern.

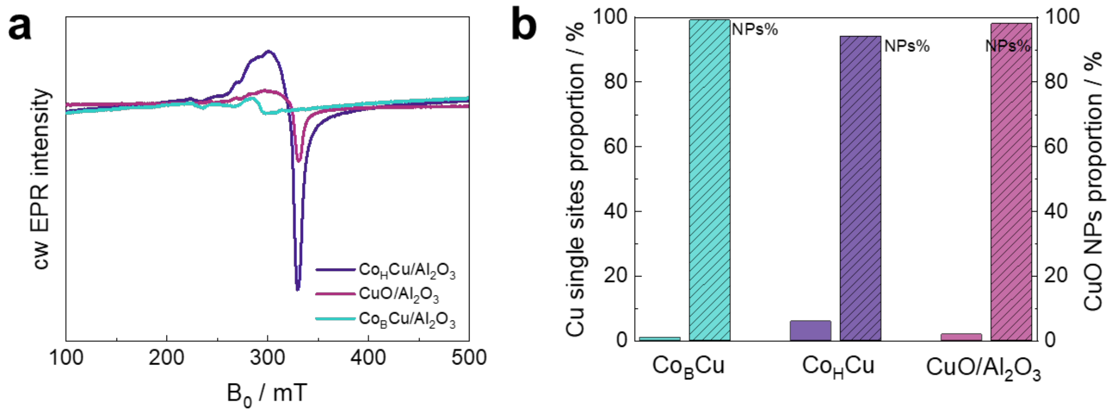

Fig. S6. Electron paramagnetic resonance (EPR) spectra. a EPR spectra of Co_BCu/Al_2O_3, Co_HCu/Al_2O_3 and CuO/Al_2O_3; **b** proportion of Cu single sites and CuO nanoparticles.

EPR spectroscopy: Experiments were performed in continuous-wave (cw) mode on a Bruker E580 X-band EPR spectrometer equipped with a Bruker ER4122-SHQE cavity. Then, 20 mg powder of each sample was loaded into a high-purity quartz EPR tube (4.0 mm o.d., 3.0 mm i.d.) for measurements. All cw EPR spectra were acquired at room temperature over a wide magnetic field range. Typical spectrometer parameters were sweep time (300 s), center field (300 mT), sweep width (300 mT), modulation frequency (100 kHz), microwave frequency (9.87 GHz), and microwave power (2.0 mW).

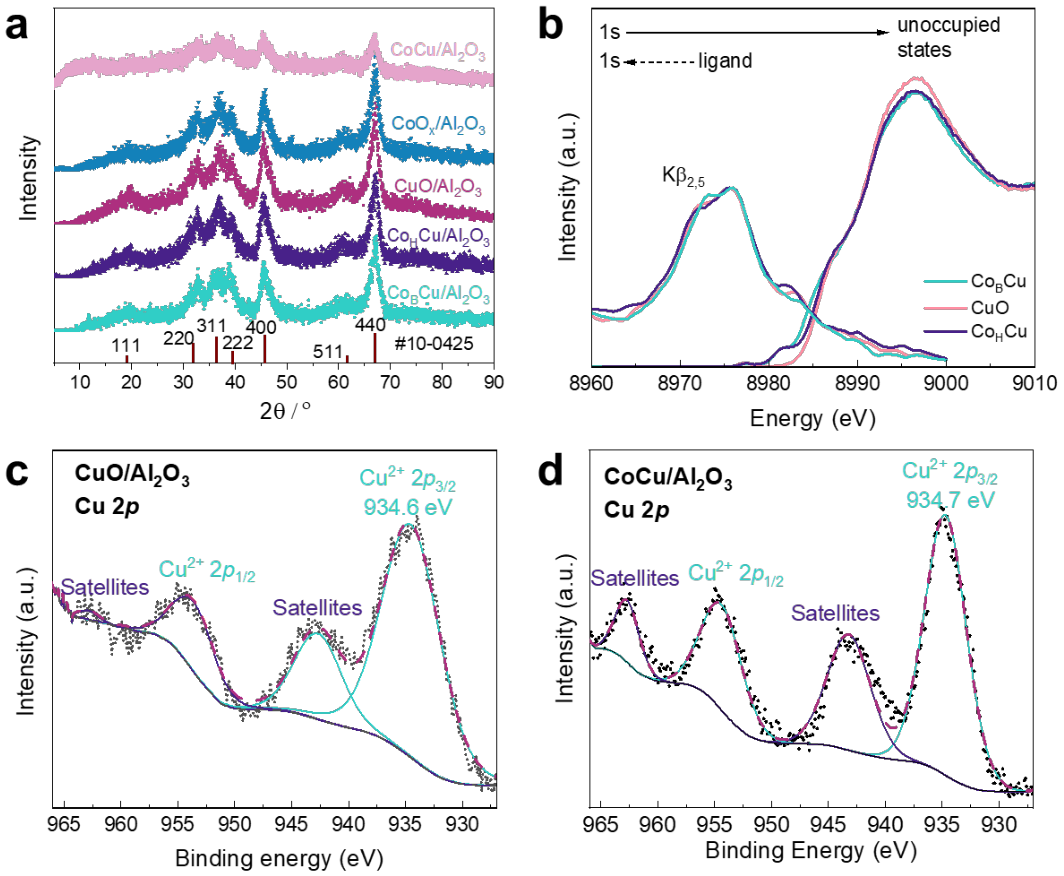

Fig. S7. X-Ray diffraction (XRD) patterns and XPS spectra. a XRD patterns of CoO_X/Al_2O_3, CuO/Al_2O_3, Co_BCu/Al_2O_3 and Co_HCu/Al_2O_3. JCPDS card number of γ-Al_2O_3: #10-0425 (Peaks at $2\theta = 19.2$, 32.0, 36.6, 39.3, 46, 61.5 and 67°, are indexed as the (111), (220), (311), (222), (400), (511) and (440) reflections of γ-Al_2O_3.); **b** VtC and XANES of CuO/Al_2O_3, Co_BCu/Al_2O_3 and Co_HCu/Al_2O_3; Cu 2*p* XPS spectra of CuO/Al_2O_3 (**c**) and $CoCu/Al_2O_3$ (**d**).

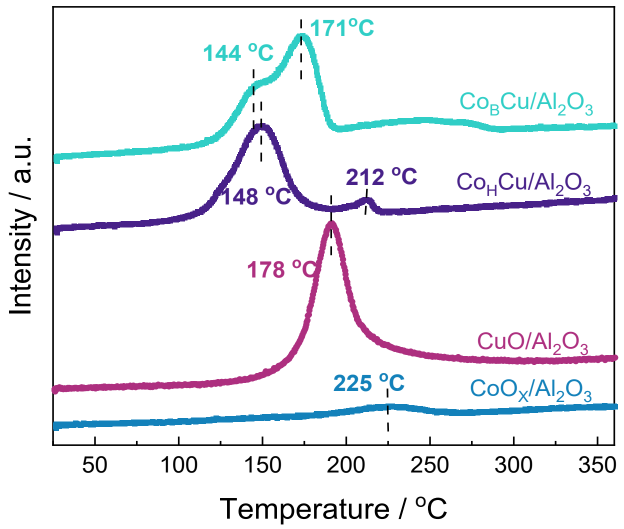

Fig. S8. Hydrogen temperature-programmed reduction (H_2-TPR). H_2-TPR of CoO_X/Al_2O_3, CuO/Al_2O_3, Co_BCu/Al_2O_3 and Co_HCu/Al_2O_3.

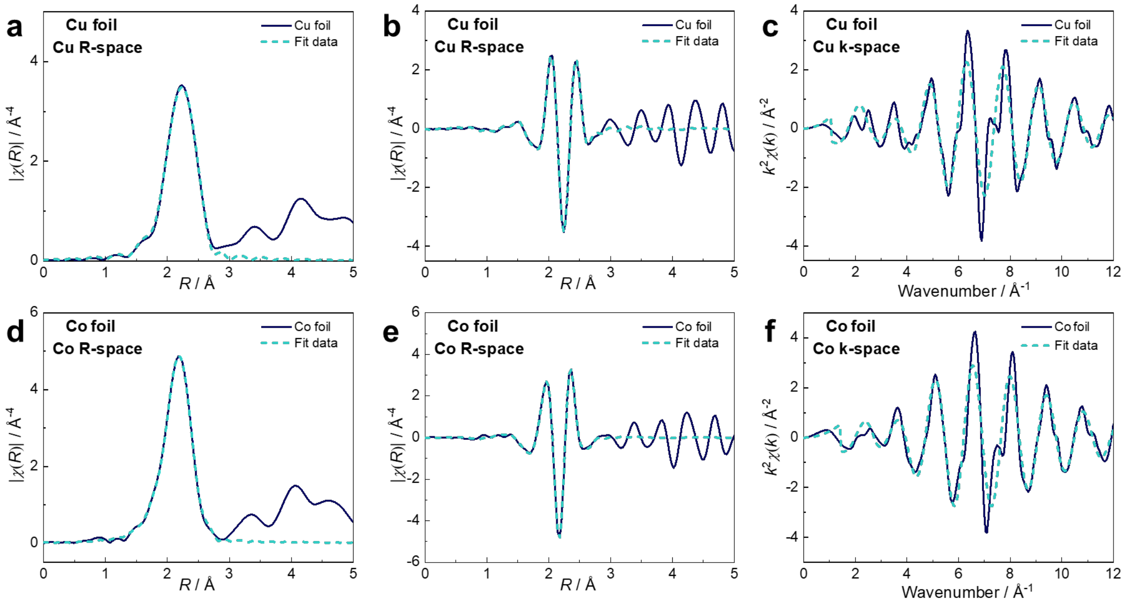

Fig. S9. EXAFS fitting of Cu foil and Co foil. EXAFS fitting results of Cu K-edge of Cu foil (**a-c**) and Co K-edge of Co foil (**d-f**) in R-space, R-space (without phase correction) and k-space.

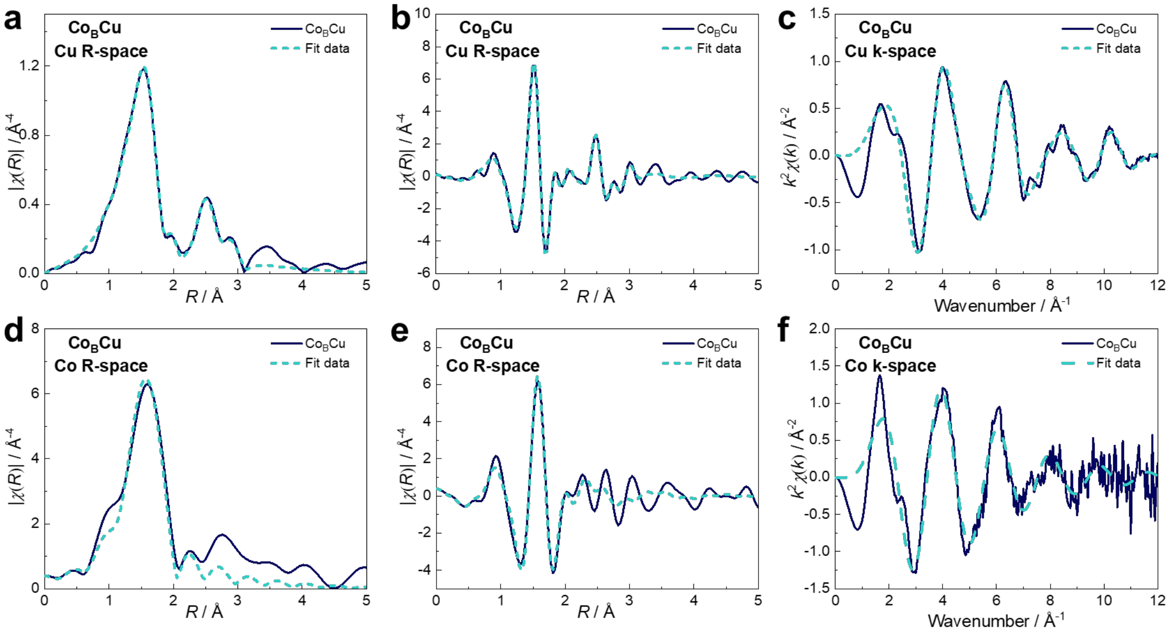

Fig. S10. EXAFS fitting of Co_BCu/Al_2O_3. EXAFS fitting results of Cu K-edge (**a-c**) and Co K-edge (**d-f**) of Co_BCu/Al_2O_3 in R-space, R-space (without phase correction) and k-space.

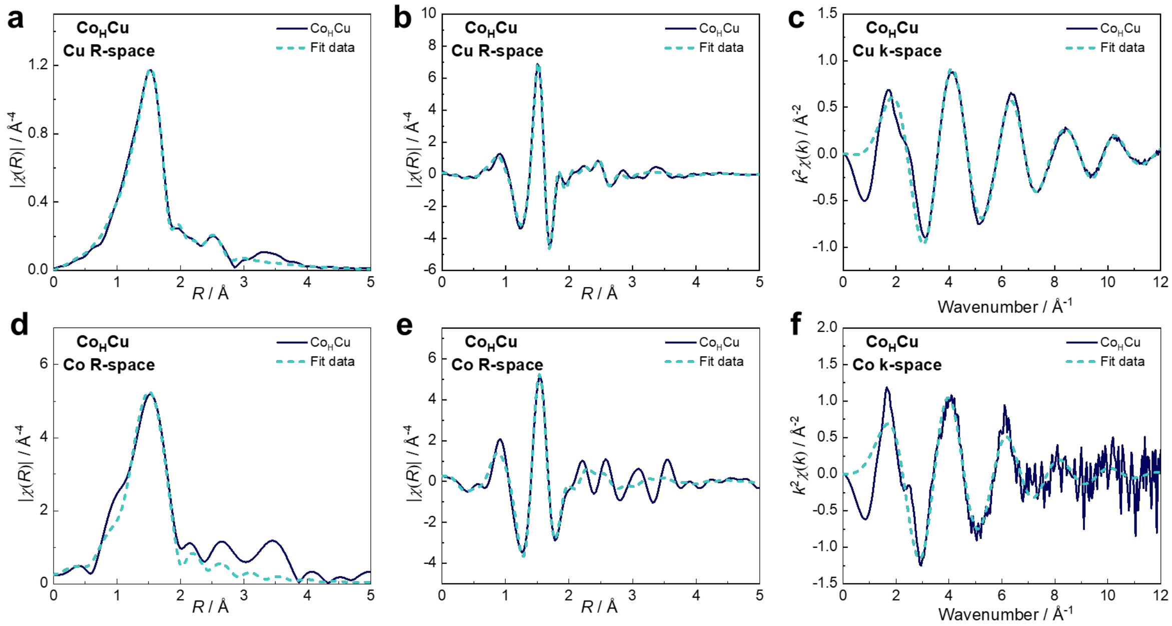

Fig. S11. EXAFS fitting of Co_HCu/Al_2O_3. EXAFS fitting results of Cu K-edge (**a-c**) and Co-K edge (**d-f**) and Co_HCu/Al_2O_3 in R-space, R-space (without phase correction) and k-space.

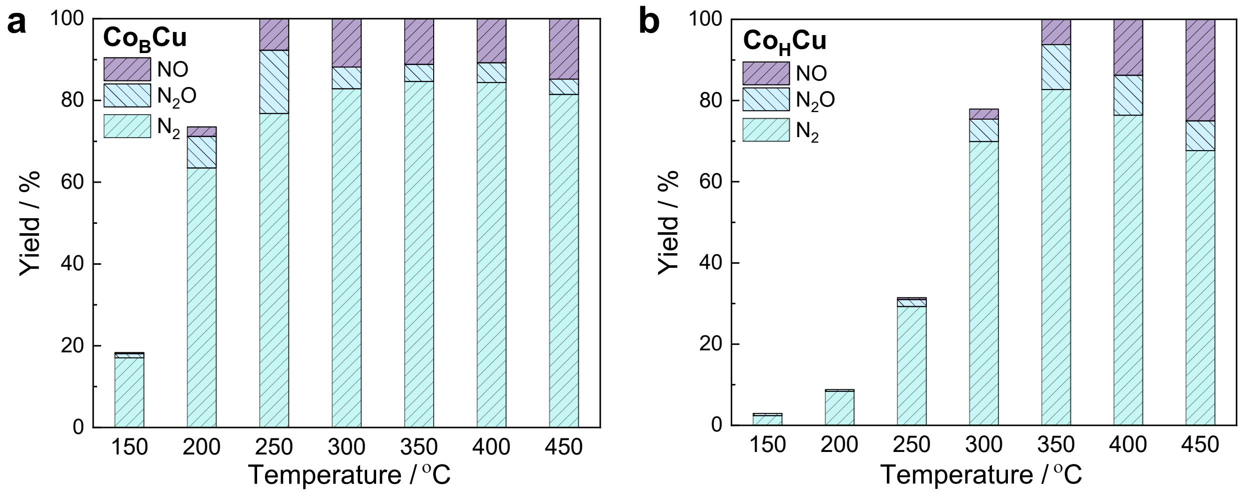

Fig. S12. Performance of the catalysts in the oxidation of NH_3 at different temperatures. a Co_BCu/Al_2O_3 and **b** Co_HCu/Al_2O_3. (Reaction conditions: 50 mg catalyst, 5000 ppm NH_3, 5% O_2 balanced in He, gas flow: 100 mL/min).

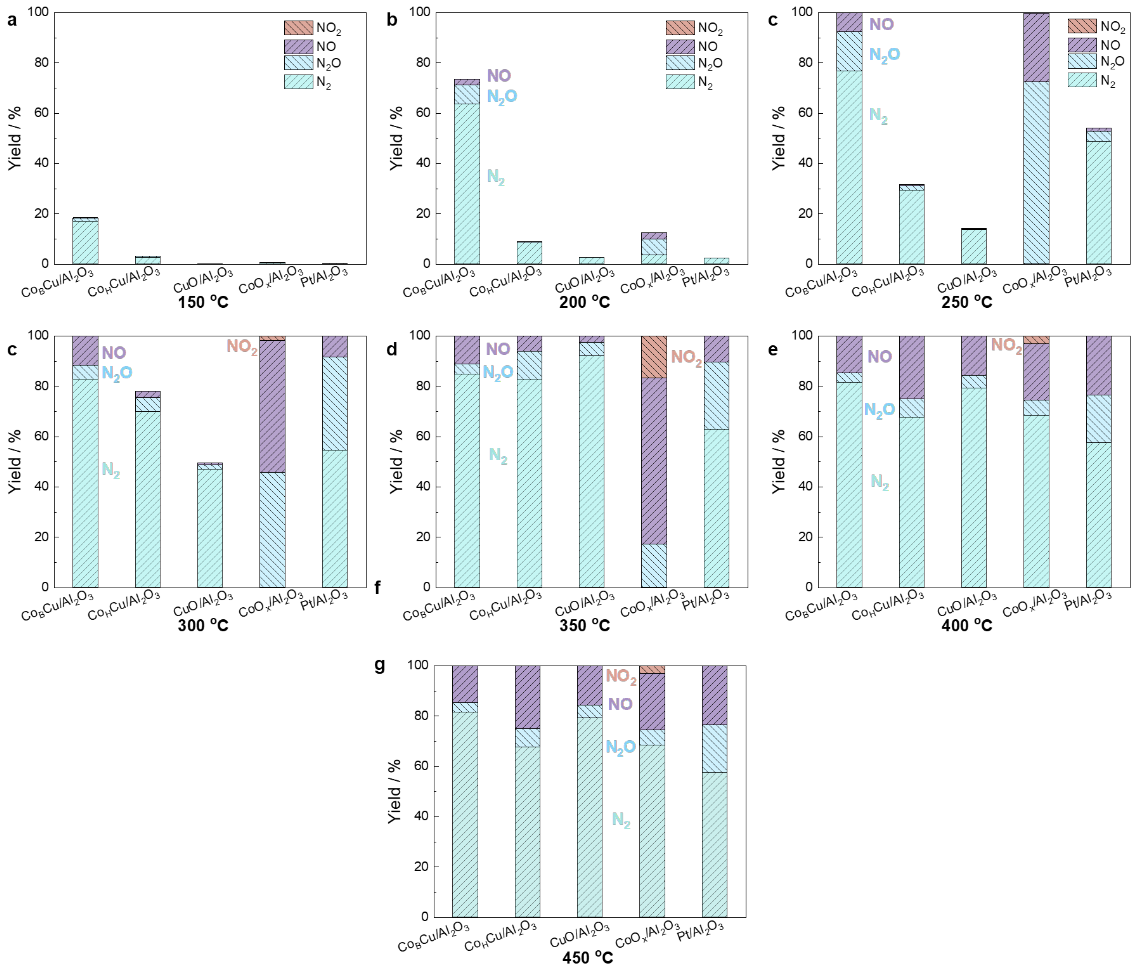

Fig. S13. Performance of the catalysts in the oxidation of NH_3 at different temperatures. a 150 ℃, **b** 200 ℃, **c** 250 ℃, **d** 300 ℃, **e** 350 ℃, **f** 400 ℃, **g** 450 ℃. (Reaction conditions: 50 mg catalyst, 5000 ppm NH_3, 5% O_2 balanced in He, gas flow: 100 mL/min).

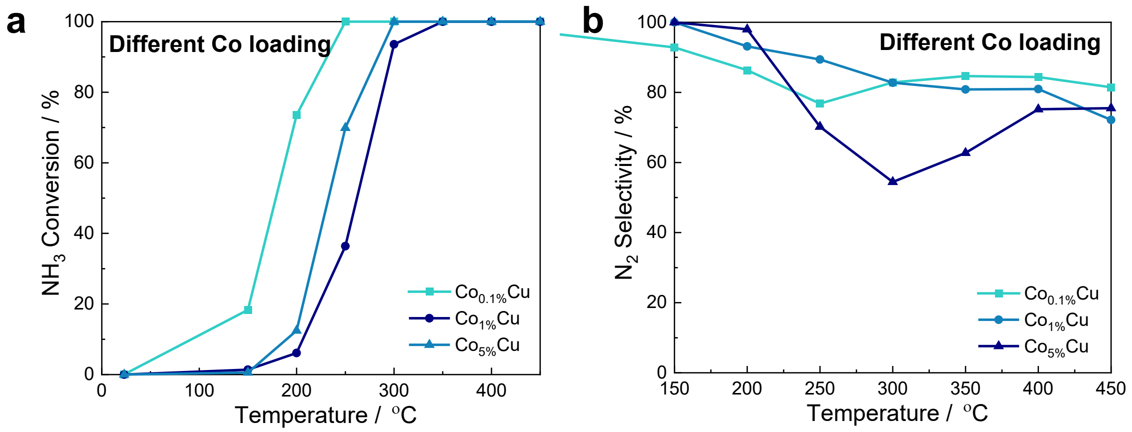

Fig. S14. Performance of Co_BCu/Al_2O_3 with different Co loading in the oxidation of NH_3 at different temperatures. a Conversion of NH_3, **b** selectivity to N_2. (Reaction conditions: 50 mg catalyst, 5000 ppm NH_3, 5% O_2 balanced in He, gas flow: 100 mL/min).

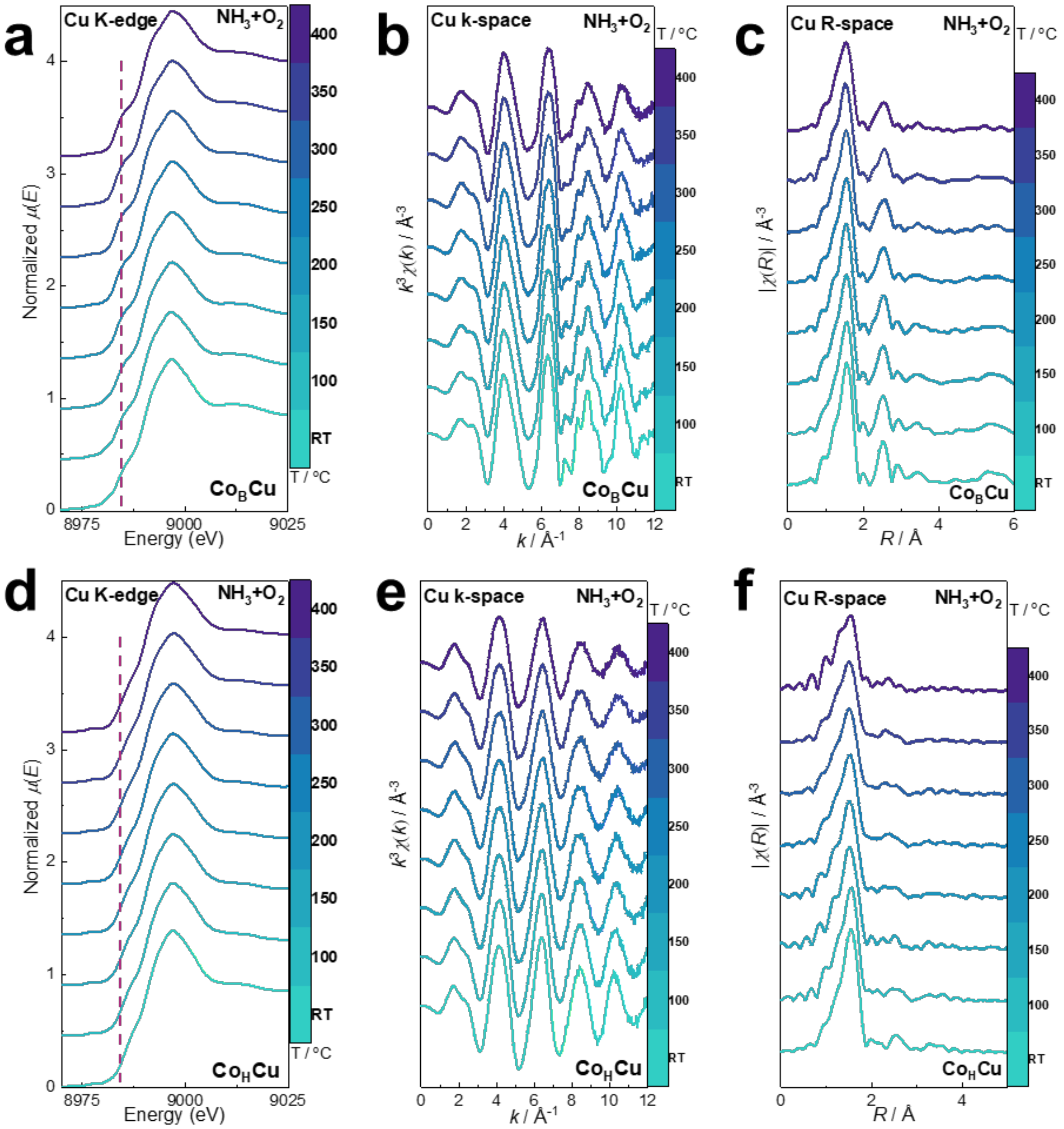

Fig. S15. ***Operando*** **Cu K-edge EXAFS spectra at different reaction temperatures.** Operando EXAFS Cu K-edge, k-space and R-space of Co_BCu/Al_2O_3 (**a, b, c**) and Co_HCu/Al_2O_3 (**d, e, f**) as a function of temperature.

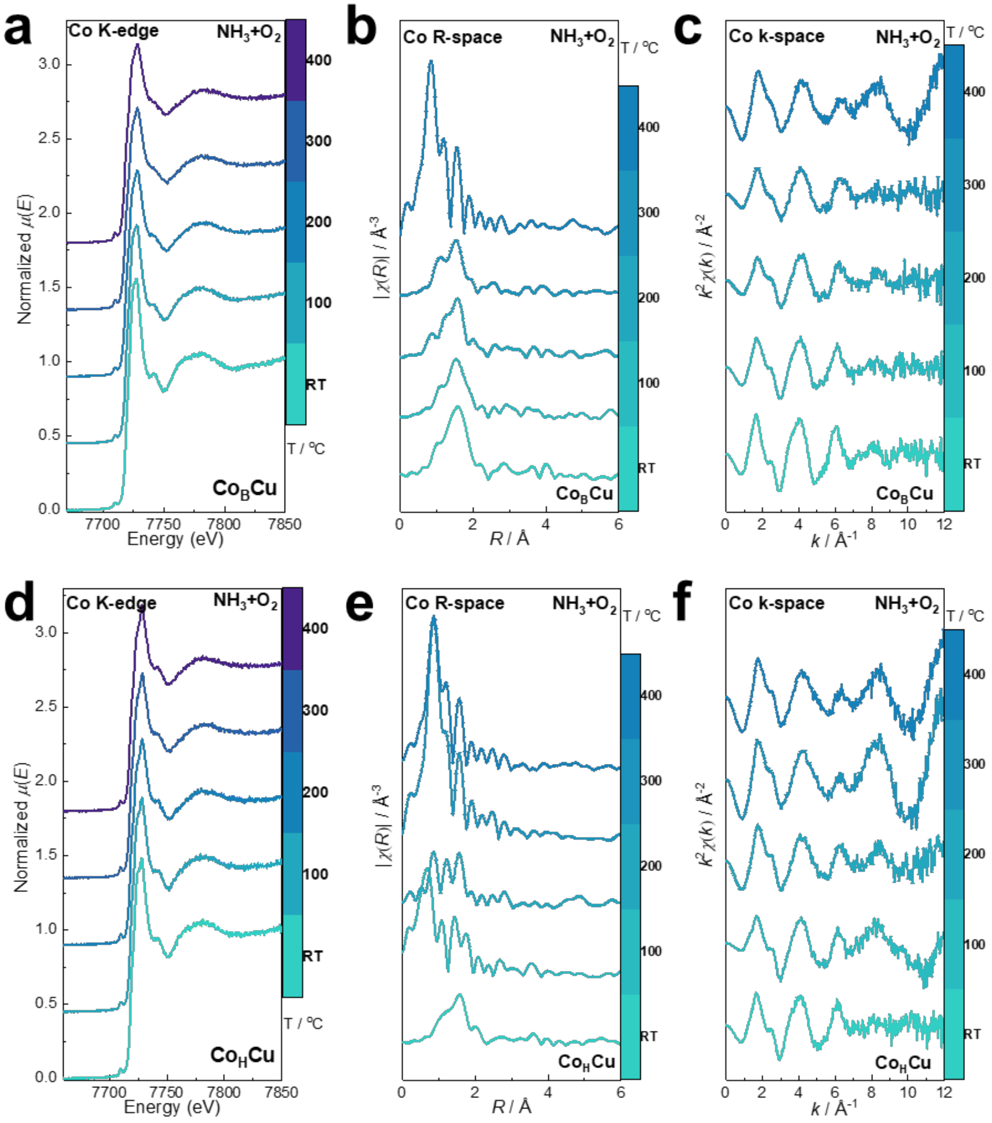

Fig. S16. *Operando* Co K-edge EXAFS spectra at different reaction temperatures. Operando EXAFS Co K-edge, R-space and k-space of Co_BCu/Al_2O_3 (**a, b, c**) and Co_HCu/Al_2O_3 (**d, e, f**) as a function of temperature.

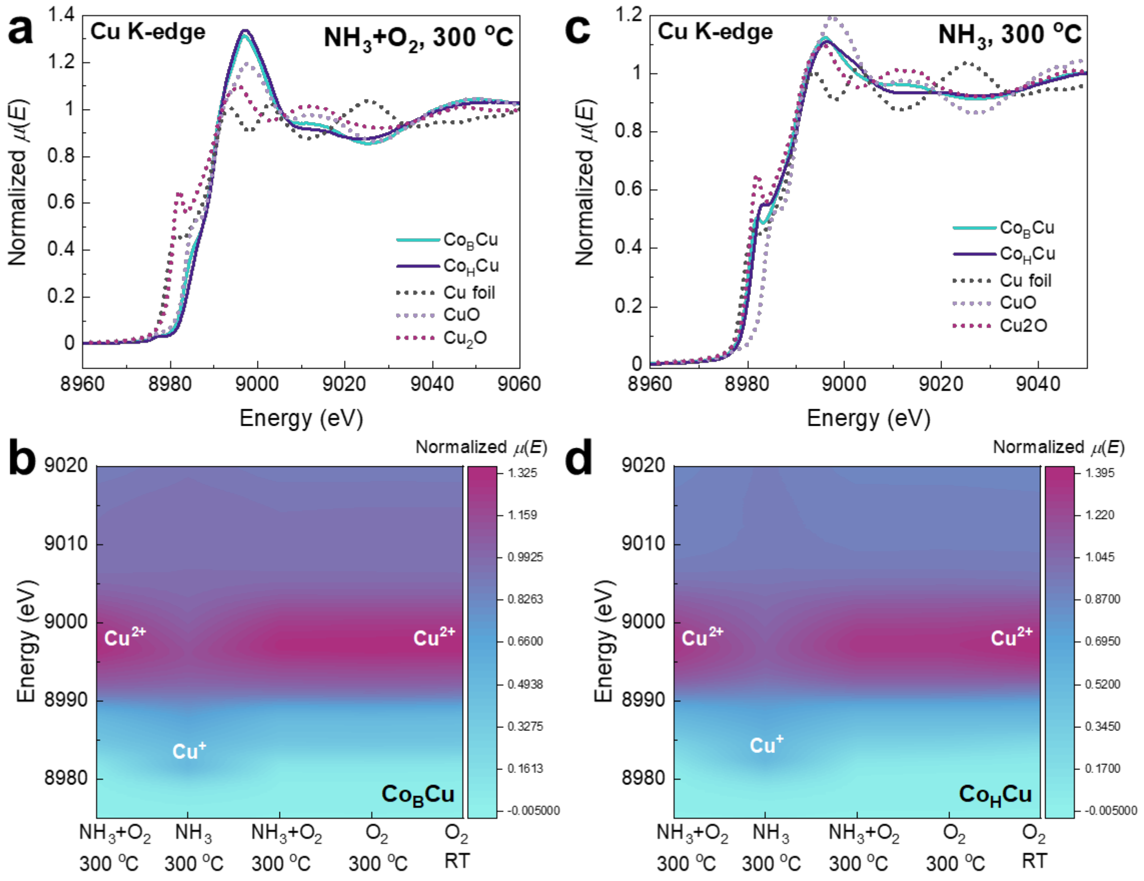

Fig. S17. ***Operando*** **Cu K-edge XANES spectra of different catalysts under different gases.** Operando XANES Cu K-edge of Co_BCu/Al_2O_3 and Co_HCu/Al_2O_3 in NH_3+O_2 at 300 °C (**a**) or in NH_3+O_2 at 300 °C (**b**); 2D XAFS maps of Co_BCu/Al_2O_3 (**c**) and Co_HCu/Al_2O_3 (**d**) in different gases.

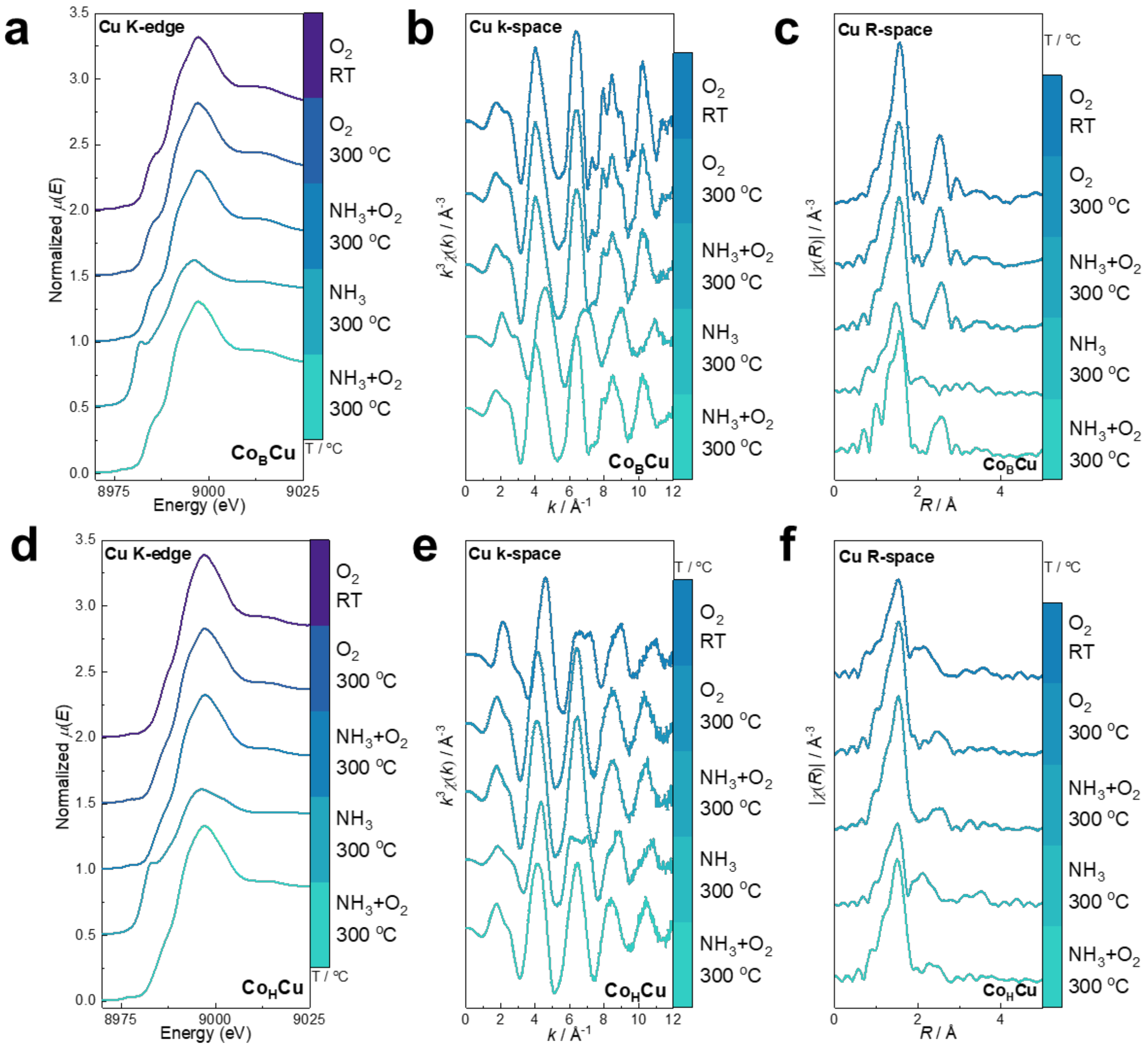

Fig. S18. ***Operando* Cu K-edge EXAFS spectra in different gases.** In-situ EXAFS Cu K-edge, k-space and R-space of Co_BCu/Al_2O_3 (**a, b, c**) and Co_HCu/Al_2O_3 (**d, e, f**) in different gases.

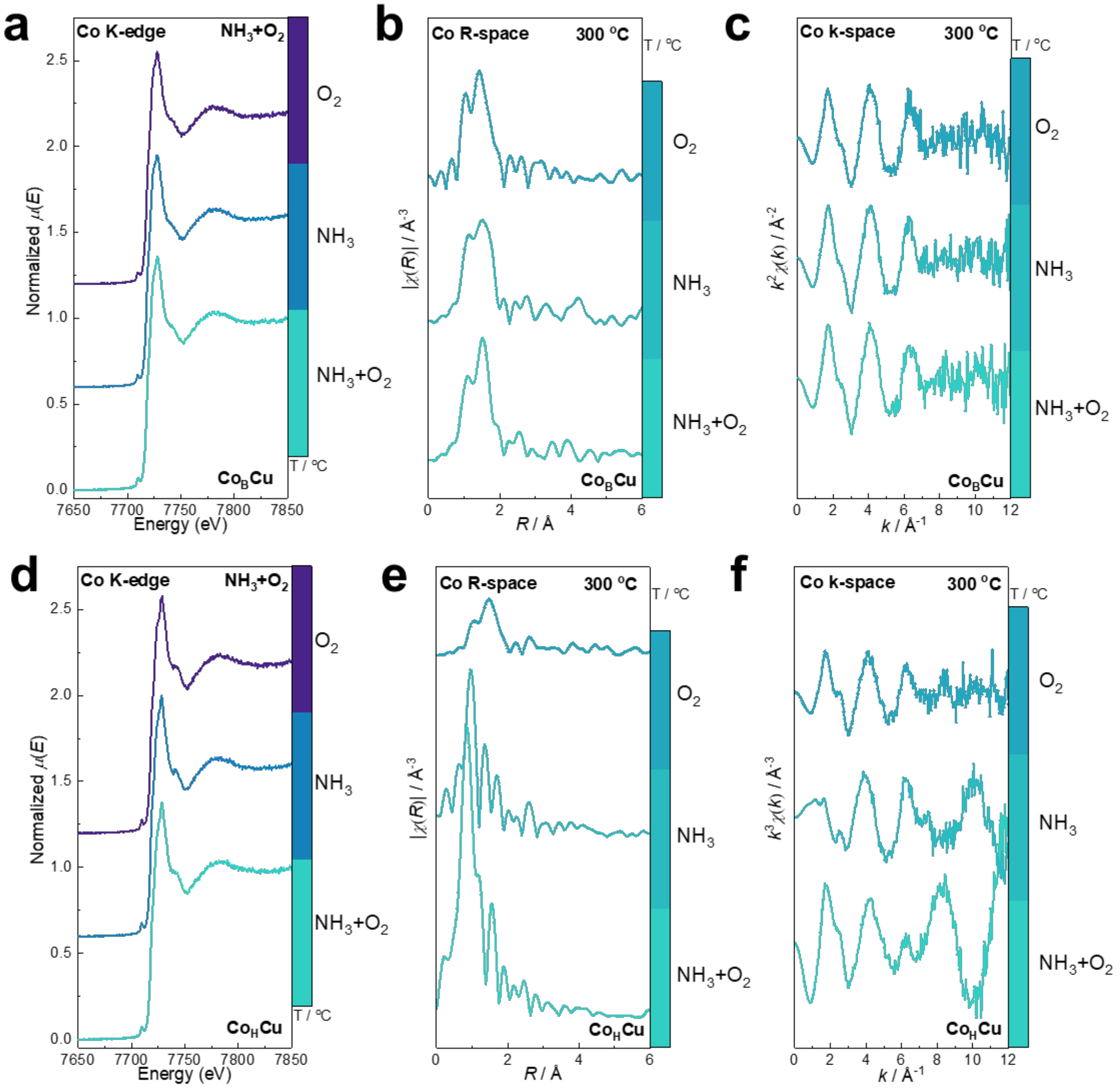

Fig. S19. ***Operando*** **Co K-edge EXAFS spectra in different gases.** Operando EXAFS Co K-edge, R-space and k-space of Co_BCu/Al_2O_3 (**a, b, c**) and Co_HCu/Al_2O_3 (**d, e, f**) in different gases.

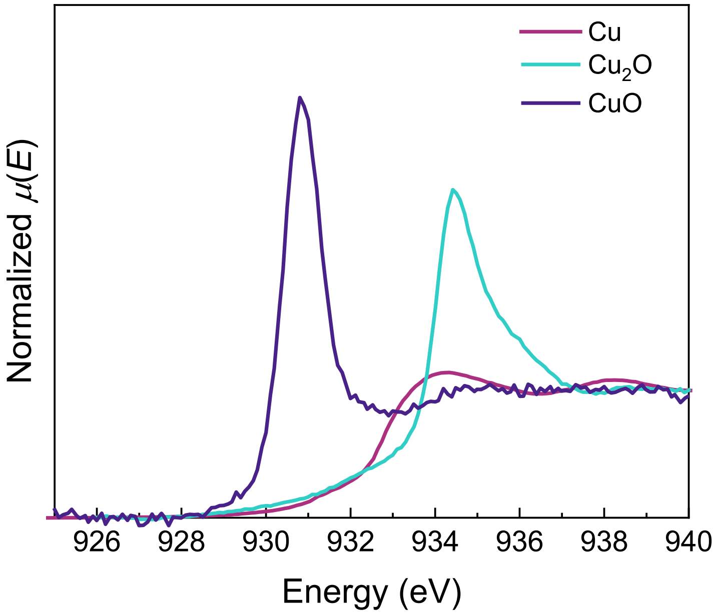

Fig. S20. Cu L-edge of Cu foil, Cu_2O and CuO. It is in Auger electron yield (AEY) mode.

Table S1. EXAFS fitting results.

Sample	Scattering	C.N.	d (Å)	σ^2	R-factor	E_0 (eV)
Cu foil STD	Cu-Cu	12	2.56			
Cu_2O STD	Cu-O	2	1.85			
	Cu-Cu	12	3.01			
CuO STD	Cu-O	4	1.95			
	Cu-Cu	4	2.88			
		4	3.07			
		2	3.16			
Co foil STD	Co-Co	12	2.50			
Co_BCu/Al_2O_3	Cu-O	3.45 ± 0.12	1.96± 0.005	0.005 ± 0.0005	0.003	-0.42 ± 0.42
	Cu-Cu(1)	2.39 ± 0.48	2.91 ± 0.03	0.009 ± 0.002		
	Cu-Cu(2)	1.90 ± 0.39	3.10 ± 0.03	0.009 ± 0.002		
Co_BCu/Al_2O_3	Co-O	5.74 ± 0.99	2.04± 0.0002	0.008 ± 0.003	0.013	-0.51 ± 1.97
	Co-Co	-	-	-		
Co_HCu/Al_2O_3	Cu-O	3.33 ± 0.12	1.93 ± 0.003	0.005 ± 0.0005	0.004	-0.77 ± 0.50
	Cu-Cu (1)	0.22 ± 0.21	2.91 ± 0.021	0.005 ± 0.007		
	Cu-Cu (2)	-	-	-		
Co_HCu/Al_2O_3	Co-O	5.92 ± 1.09	1.99 ± 0.004	0.010 ± 0.003	0.019	-2.95 ± 2.14
	Co-Co	-	-	-		
